# Addressing voltage decay in Li-rich cathodes by broadening the gap between metallic and anionic bands

**DOI:** 10.1038/s41467-021-23365-9

**Published:** 2021-05-24

**Authors:** Jicheng Zhang, Qinghua Zhang, Deniz Wong, Nian Zhang, Guoxi Ren, Lin Gu, Christian Schulz, Lunhua He, Yang Yu, Xiangfeng Liu

**Affiliations:** 1 Center of Materials Science and Optoelectronics Engineering, College of Materials Science and Optoelectronic Technology, University of Chinese Academy of Sciences, Beijing, P. R. China; 2 Beijing National Laboratory for Condensed Matter Physics, Institute of Physics, Chinese Academy of Science, Beijing, P. R. China; 3 Department of Dynamics and Transport in Quantum Materials, Helmholtz-Center Berlin for Materials and Energy, Hahn-Meitner-Platz 1, Berlin, Germany; 4 Shanghai Institute of Microsystem and Information Technology, Chinese Academy of Sciences, Shanghai, P. R. China; 5 Songshan Lake Materials Laboratory, Dongguan, P. R. China; 6 Spallation Neutron Source Science Center, Dongguan, P. R. China; 7 CAS Center for Excellence in Topological Quantum Computation, University of Chinese Academy of Sciences, Beijing, China

**Keywords:** Batteries, Batteries, Batteries

## Abstract

Oxygen release and irreversible cation migration are the main causes of voltage fade in Li-rich transition metal oxide cathode. But their correlation is not very clear and voltage decay is still a bottleneck. Herein, we modulate the oxygen anionic redox chemistry by constructing Li_2_ZrO_3_ slabs into Li_2_MnO_3_ domain in Li_1.21_Ni_0.28_Mn_0.51_O_2_, which induces the lattice strain, tunes the chemical environment for redox-active oxygen and enlarges the gap between metallic and anionic bands. This modulation expands the region in which lattice oxygen contributes capacity by oxidation to oxygen holes and relieves the charge transfer from anionic band to antibonding metal–oxygen band under a deep delithiation. This restrains cation reduction, metal–oxygen bond fracture, and the formation of localized O_2_ molecule, which fundamentally inhibits lattice oxygen escape and cation migration. The modulated cathode demonstrates a low voltage decay rate (0.45 millivolt per cycle) and a long cyclic stability.

## Abstract (web summary)

Voltage fade is a critical issue for Li-rich transition metal oxide cathode. Here, the authors modulate the oxygen anionic redox chemistry and enlarges the gap between metallic and anionic states by constructing Li_2_ZrO_3_ slabs into Li_2_MnO_3_ domain in Li_1.21_Ni_0.28_Mn_0.51_O_2_ which fundamentally suppresses the voltage decay.

## Introduction

Increasing dependence of electric vehicles and energy-storage systems on high-energy Li-ion batteries pressingly calls for continual developments in the performance of cathode materials. The improvement space for traditional cathode materials based on transition metal (M) cationic redox is limited to the gradually approached energy-density ceiling^1^. Such limitation is foreseen to be transcended by Li-rich Mn-based oxides, which exhibit both M and O redox activities and a high reversible capacity (>250 mA h g^−1^)^2^. But Li-rich Mn-based oxides universally manifest a daunting challenge of voltage fade with electrochemical cycling, seriously obstructing its practical applications. Although a variety of methods, including bulk doping^3^, surface modification^4^, and adjusting element compositions^5,6^, have been adopted to restrain voltage fade, none can fundamentally solve it. Recently, scientists have achieved remarkable results in suppressing voltage fade using some other strategies^7–11^. For example, Li et al. prepared Li-gradient Li-rich single crystals by extracting LiO from molten salt, showing a voltage attenuation rate of 1.17 mV per cycle^7^. Kang’s group synthesized an O2-type Li-rich oxide by ion-exchanging, exhibiting a voltage decay rate of about 1.1 mV per cycle^9^. However, the voltage fade issue for Li-rich oxides has still not been fundamentally solved, and its underlying mechanism remains unclear. Both experiments and calculations demonstrate that the direct cause of voltage fade is the irreversible migration of M cations into an empty Li site and the subsequent structural collapse^12–16^. Recent research manifests that M migration is intrinsically linked with oxygen anionic redox (OAR)^17^. For thoroughly studying the voltage decay mechanism, and better suppressing voltage attenuation, a substantial key should lie in regulating OAR chemistry. But this is slowed down by two difficulties.

First, accurate regulation of specific Li_2_MnO_3_ lattice should be achieved. The OAR activities are originated from O 2*p* lone pairs (׀O_2*p*_). The key of regulating OAR chemistry is to modulate the chemical environment of lattice oxygen with redox activity, which determines the OAR characteristics^18^. The structure of Li-rich Mn-based oxides is complicated with both hexagonal LiMO_2_ and rhombic Li_2_MnO_3_ structures^19^. The rhombic Li_2_MnO_3_ domain, where OAR reaction occurs, is difficult to realize the precise modulation with current modification strategies. Recently, the cation-disordered rock-salt structure has been shown to be positive on stabilizing OAR chemistry^20,21^. Nevertheless, the preparation procedures of these strategies are tedious, which are hard to precisely regulate the lattice of rhombic Li_2_MnO_3_ in the complicated Li-rich Mn-based oxides. Therefore, explorations of simple, economical, and feasible modifications to precisely modulate Li_2_MnO_3_ lattice are highly required.

Second, OAR chemistry should be deeply understood. The chemistry of OAR is very complex, and the understanding of OAR is still quite insufficient. In recent years, scientists have made some breakthroughs on OAR from revealing its activity origin^22^ to exposing the chemical kinetics and chemical thermodynamic properties of OAR reactions^23^. But current knowledge still cannot explain the differences in OAR chemistry of Li-rich oxides with different metals and subtle structural properties. For example, lattice oxygen in 4*d*/5*d* M-based Li-rich oxides evolves into O–O dimer as the oxidation product^24^, while the oxygen oxidation product for 3*d* M-based Li-rich oxides is detected as O–O dimer or lattice oxygen with an electron hole by different groups^22,25^. Recently, House et al. reported that lattice oxygen in alkali-rich transition metal oxide is oxidized to localized oxygen molecules during charging, and Mn migration occurs at the same time^26^. A coupling mechanism is proposed based on the experimental and calculated results that the formation of peroxide is stabilized by M–O decoordination and cation migration^27^. But some researchers declared that cation migration should be caused by OAR instability rather than a coupling phenomenon of OAR^28,29^. These uncertainties and divergences are the critical issues that prevent OAR from being fully understood and hinder voltage fade from being fundamentally resolved.

In this work, we construct Li_2_ZrO_3_ slabs into the lattice of Li_2_MnO_3_ domain in a Li-rich Mn-based oxide that induces lattice strain due to the lattice differences between Li_2_ZrO_3_ and Li_2_MnO_3_, and modulates the chemical environment for redox-active oxygen and OAR chemistry. Mn_2_Li slabs undergo a relative slip of ~3°, with Mn–O bond being elongated. Experimental and calculated results indicate that the regulated structure modifies the chemical environment of lattice oxygen, and broadens the gap between metallic and anionic bands. This modulation simultaneously expands the region in which lattice oxygen contributes capacity by oxidation to oxygen holes, and relieves the surpassing of the anionic state above the metallic state and the charge transfer from anionic band to (M–O)* band. This inhibits the fracture of active lattice O–M bond, the reduction of M cations, and the formation of localized O_2_ molecule, which fundamentally restrains oxygen release and cation migration. The improved cathode demonstrates a high discharge capacity (264 mAh g^−1^), a high average voltage (3.65 V), and a record low-voltage decay rate (0.45 mV per cycle). This study discloses the crucial role of energy band modulation in designing anionic redox-active cathode materials with high performances.

## Results

### Constructing Li_2_ZrO_3_ slab into Li_2_MnO_3_ domain in Li-rich oxide cathode

**Fig. 1:**
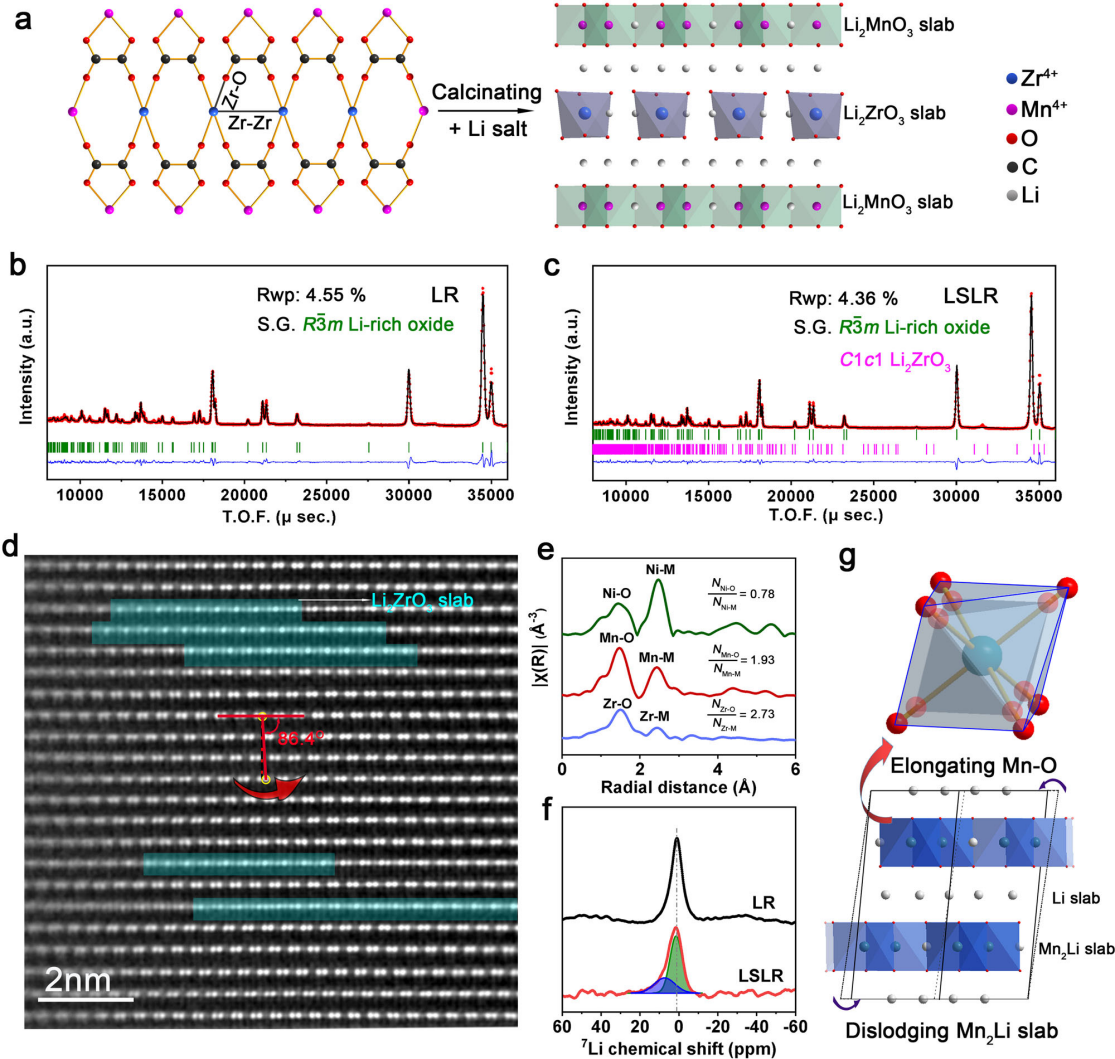
Structural properties of LSLR. **a** Schematic procedure to synthesize LSLR. **b**, **c** Rietveld refined neutron diffraction patterns of LR (**b**) and LSLR (**c**). **d** AC-STEM image of LSLR observed along the [110] axis. **e** Fourier-transformed Ni/Mn/Zr K-edge EXAFS. *N* is the coordination number. **f** ^7^Li MAS-NMR spectra for LR and LSLR. **g** Schematic illustration of the proposed structure for the Li_2_MnO_3_ domain in LSLR. Source data are provided as a Source Data file.

In this study, we took Li_1.21_Ni_0.28_Mn_0.51_O_2_ as a typical example of Li-rich Mn-based oxides to explore. Feasible precipitation–calcination route is adopted to introduce Li_2_ZrO_3_ slabs into Li_2_MnO_3_ domain in Li-rich oxide (LSLR) and a referenced Li-rich oxide (LR) is also synthesized. Inductively coupled plasma optical emission spectrometer (ICP-OES, Supplementary Table 1) has confirmed that the molecular formulas of LR and LSLR are Li_1.21_Ni_0.28_Mn_0.51_O_2_ and Li_1.21_Ni_0.28_Mn_0.49_Zr_0.02_O_2_, respectively. The key to the synthesis of LSLR is the selection of oxalate and Zr^4+^ as precipitator and Zr source, respectively. The structures for precursors of LSLR are revealed by X-ray diffraction (XRD), X-ray absorption spectroscopy (XAS), and the wavelet transform of XAS spectra (Supplementary Figs. 1–3). The results indicate that a linear Zr arrangement is formed in LSLR precursor as illustrated in Fig. 1a. After lithiation, Zr^4+^ arrays in the precursor evolve into Li_2_ZrO_3_ slabs, and the Mn^4+^ adjacent to the Zr^4+^ arrays evolves into Li_2_MnO_3_ domain. The synthesis diagram of LSLR is declared in Fig. 1a.

The structural properties of LSLR are characterized by XRD and neutron diffraction (ND) Rietveld refinement (Supplementary Fig. 4 and Fig. 1b, c). XRD plots declare that both LR and LSLR follow the lattice frame of Li-rich layered oxides, except for a weak peak assigned to Li_2_ZrO_3_ observed in LSLR. The neutron diffraction Rietveld refinement results (Supplementary Table 2) show that, compared with LR, parameters *a* and *c* of LSLR were both larger, the octahedron of MnO_6_ was obviously enlarged, and the Mn–O bond length was extended from 1.918 to 1.946 Å. SEM images demonstrate that no obvious morphology difference between LR and LSLR is observed (Supplementary Fig. 5).

Spherical aberration-corrected scanning transmission electron microscopy (AC-STEM) image of LSLR along [110] axis of Li_2_MnO_3_ domain is provided in Fig. 1d. Two atomic arrangements are exhibited by LSLR, with most atoms arranged in accordance with M–M–Li rule and a few in accordance with M–Li–Li rule, which belong to Li_2_MnO_3_ and Li_2_ZrO_3_ slabs, respectively. This is also verified by the line-intensity profiles (Supplementary Fig. 6) and extended X-ray absorption fine- structure (EXAFS) spectra (Fig. 1e and Supplementary Fig. 7). As declared by the crystal structures of Li_2_MnO_3_ and Li_2_ZrO_3_ in Supplementary Fig. 8, the metal cations in Li_2_MnO_3_ are arranged alternately by Li/Mn layer and Li layer, while in Li_2_ZrO_3_, only Li/Zr layer is stacked.

Here in our samples, it is worth noting that the stacking sequence of Mn_2_Li plate is largely affected. Compared with the perfect Li_2_MnO_3_ lattice (Supplementary Fig. 9), the Mn_2_Li layer of LSLR has a relative slip of ~3°. This change is attributed to the presence of Li_2_ZrO_3_ slab distributed in the Mn_2_Li plate of Li_2_MnO_3_ and the resulted lattice strain between Li_2_ZrO_3_ and Li_2_MnO_3_. To confirm this, AC-STEM was also performed on LR (Supplementary Fig. 10). The result showed that the Li_2_MnO_3_ lattice in LR matched perfectly the lattice arrangement of Li_2_MnO_3._ The chemical environments of Li are characterized by ^7^Li magic-angle spinning (MAS) nuclear magnetic resonance (NMR) spectra. Compared with LR, MAS-NMR results declare that the chemical environment of 30.7% Li in LSLR is changed. These data are far greater than the added Zr content (2%), which should be caused by Mn_2_Li plate stacking sequence changing, evidenced by STEM image in Fig. 1f. Based on the above analysis, the crystal structure of Li_2_MnO_3_ domain in LSLR is confirmed to be slightly distorted as shown in Fig. 1g.

### Electrochemical performances of LSLR

**Fig. 2:**
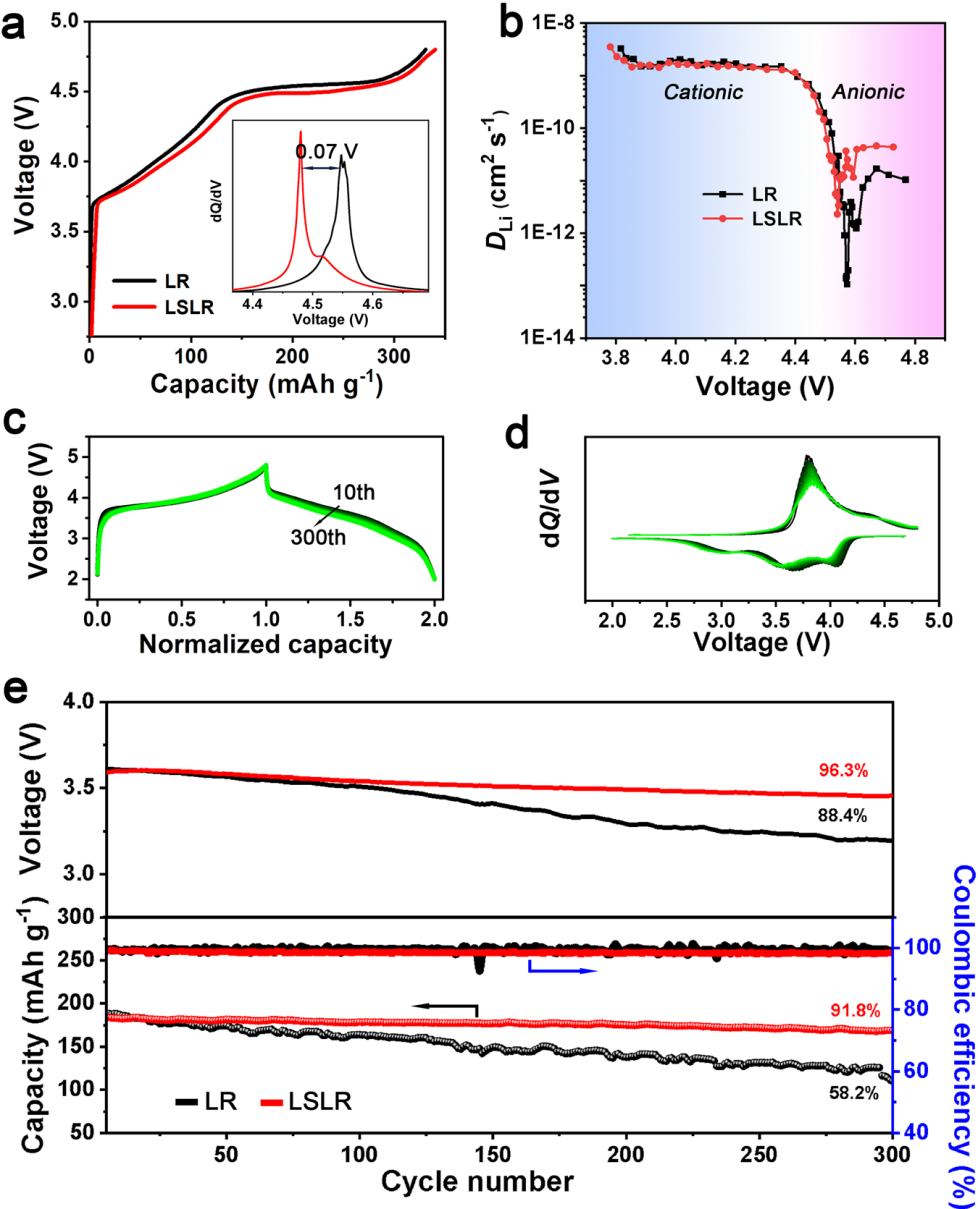
Electrochemical characterizations of LR and LSLR. **a** The initial charging plots for LR and LSLR. The inset displays the corresponding d*Q*/d*V* curves. **b**, The calculated Li^+^ diffusion coefficient from GITT. **c**, **d** Normalized voltage-capacity profiles for the 10th cycle (**c**) and d*Q*/d*V* curves for the 10th cycle (**d**) for LSLR, *n* is from 1 to 30. **e** Cycling performance at 1 C rate for LR and LSLR, 1 C is set as 250 mA g^−1^. Source data are provided as a Source Data file.

The initial charging plots in Fig. 2a declare that the oxygen activation plateau of LR is lower than that of LSLR. The d*Q*/d*V* plots clearly show a reduction of 0.07 V for the lattice oxygen activation threshold potential in LSLR than LR. The Li^+^ diffusion performance of LSLR is also different from that of LR. Galvanostatic intermittent titration technique (GITT, Fig. 2b and Supplementary Fig. 11) shows, at TM redox stage, the diffusion coefficient of Li^+^ (*D*_Li_) for LR and LSLR is almost at the same level. While at oxygen-activating plateau, the *D*_Li_ in LSLR is one order of magnitude higher than that in LR. These results confirm the structural differences in Li_2_MnO_3_ domain between LR and LSLR above revealed by structural characterizations. Because of the special structure, voltage and capacity attenuation of LSLR is significantly restrained. The almost unchanged shape of the charging–discharging plots (Fig. 2c) and the corresponding d*Q*/d*V* curves (Fig. 2d) of LSLR manifest a stable redox reaction and low-voltage decay rate. The voltage maintenance and galvanostatic charge/discharge performances of LR and LSLR at 1 C are presented in Fig. 2e. After 300 cycles, LSLR yields a high capacity and voltage retention (91.8% and 96.3%), while LR maintains much less (58.2% and 88.4%). The average voltage attenuation rate per cycle of LSLR is only 0.012% (0.45 mV per cycle). As far as we know, the voltage decay rate is the lowest value among all the previous reports (Supplementary Table 3). Coulomb efficiency indicates that the redox reaction in LSLR is more stable than that in LR. Moreover, LSLR also has a significant effect in suppressing voltage attenuation at a low current rate (Supplementary Fig. 12). In addition, the discharge curves of LR and LSLR are also slightly different (Supplementary Fig. 13). The above results imply different lattice oxygen redox behaviors in LR and LSLR.

### Restrained cation migration in LSLR

**Fig. 3:**
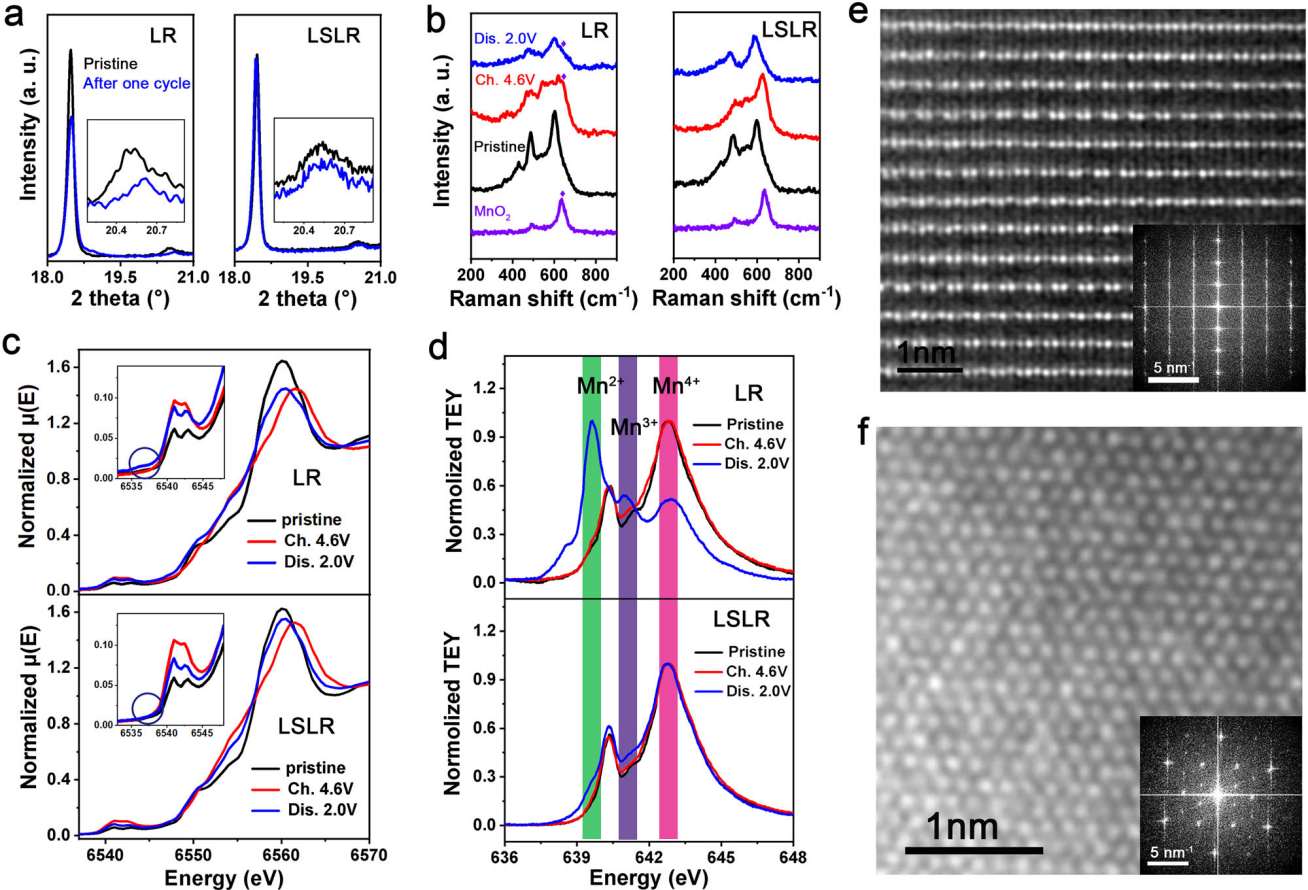
Mitigated cation migration in LSLR. **a** The XRD plots for pristine and one-cycled electrodes. The insets show the superlattice peaks. **b** Ex situ Raman results of the electrodes at different states of charge. The purple rhombus is used to label the Raman peak of MnO_2_ **c**, **d** Mn K-edge (**c**) and L_2_-edge spectra (**d**) for electrodes at different states of charge. The insets declare the pre-edge of normalized XAS spectra at Mn K-edge. Ch. and Dis. are short for charge and discharge. **e**, **f** AC-STEM and FFT images along [100] (**e**) and [103] (**f**) axis for 4.6-V charged LSLR. Source data are provided as a Source Data file.

The migration and redox behaviors of metal cations are largely influenced. Ex situ XRD plots for electrodes at different states of charge indicate that, after one cycle, LR has undergone a certain degree of structural damage, while the structure of LSLR is well maintained, which is evidenced by the coincidence degree of (003) peaks at 18.5° and superlattice peaks at 20.5° for pristine and discharge 2.0-V electrodes (Fig. 3a). Raman results in Fig. 3b demonstrate that a substance similar to rutile MnO_2_ (the crystal structure is declared in Supplementary Fig. 14) is produced on the 4.6-V charged LR electrode and does not completely disappear after full discharge, while this phenomenon is not found in the LSLR electrodes. According to the structural characteristics of Li_2_MnO_3_ and rutile MnO_2_, this MnO_2_-like product should be the result of the superlattice degradation and cation migration. XRD and Raman together demonstrate the good performance of LSLR in maintaining the superlattice.

Considering that the redox behaviors of lattice oxygen will directly affect the coordinated metal, we speculate that the above ex situ XRD and Raman distinctions are caused by the different redox evolution of Mn coordinated with active lattice oxygen during (dis)charging for LR and LSLR. To verify this, both hard and soft X-ray absorption spectra (XAS) are adopted to accurately detect the coordination and valence information of Mn cations in LR and LSLR at different electrochemical states. Two information can be obtained from Mn K-edge spectra (Fig. 3c). Compared with LR, the variations in the chemical environment and valence state of Mn in LSLR with (dis)charging are inhibited, which are evidenced by the reduced changes in the shape of the absorption edge and the white line peak intensity. The comparisons between normalized XAS spectra of the Mn K pre-edge of pristine and 4.6-V charged electrodes for LR and LSLR also support the above discussions. A faint peak, which may come from a reduced Mn–O compound, appears near the Mn K pre-edge region for the 4.6-V charged LR electrode, while LSLR does not have this new peak.

The Mn L_2_-edge spectra obtained in total electron yield (TEY) mode (Fig. 3d) reveal that, prior to charging, Mn element in LR and LSLR shows a typical lineshape for Mn^4+^, including a major peak at 642.8 eV and a weak shoulder at 640.4 eV. However, significant differences are produced after (dis)charging for Mn cations in LR and LSLR. From pristine to charged and discharged states, Mn cations in LR are increasingly reduced, declared by the appearance of peaks located at 639.6 and 641.0 eV, which are attributed to Mn^2+^ and Mn^3+^, respectively. While for Mn cations in LSLR, it is only slightly reduced at the discharged state. It indicates a different redox behavior for Mn cations in LSLR from that in LR. The restrained formation of Mn^2+^ and Mn^3+^ is favorable for inhibiting cation dissolution and maintaining structural stability^30^. In addition, the same changing trend of Ni L_3_-edge spectra with (dis)charging for LR and LSLR (Supplementary Fig. 15) rules out the possibility of the influence of Ni cations on the redox behaviors of Mn cations in two materials. Therefore, the different redox evolution of Mn cations shown by LR and LSLR is due to different oxygen redox mechanisms.

AC-STEM images of charged LSLR to 4.6 V along [100] and [103] axis of Li_2_MnO_3_ domain are provided in Fig. 3e, f. Different from the phenomenon of OAR accompanied by cation migration in Li-rich oxides reported in literature^9^, no obvious cation migration is detected in charged LSLR. It can be seen from the AC-STEM and the corresponding fast Fourier transform (FFT) images that the charged LSLR still maintains the original superlattice structure. However, for LR at the same electrochemical state, we could not find a suitable crystal direction and take clear STEM pictures, which may be due to the deteriorated structure, or generated organic decompositions on the particle surface (Supplementary Fig. 16), or great structural changes. Whatever the reason it is, the corresponding FFT image of the STEM picture for charged LR does illustrate that LR has undergone a significant structural change after charging (Supplementary Fig. 17).

### Modulated O redox chemistry of LSLR

**Fig. 4:**
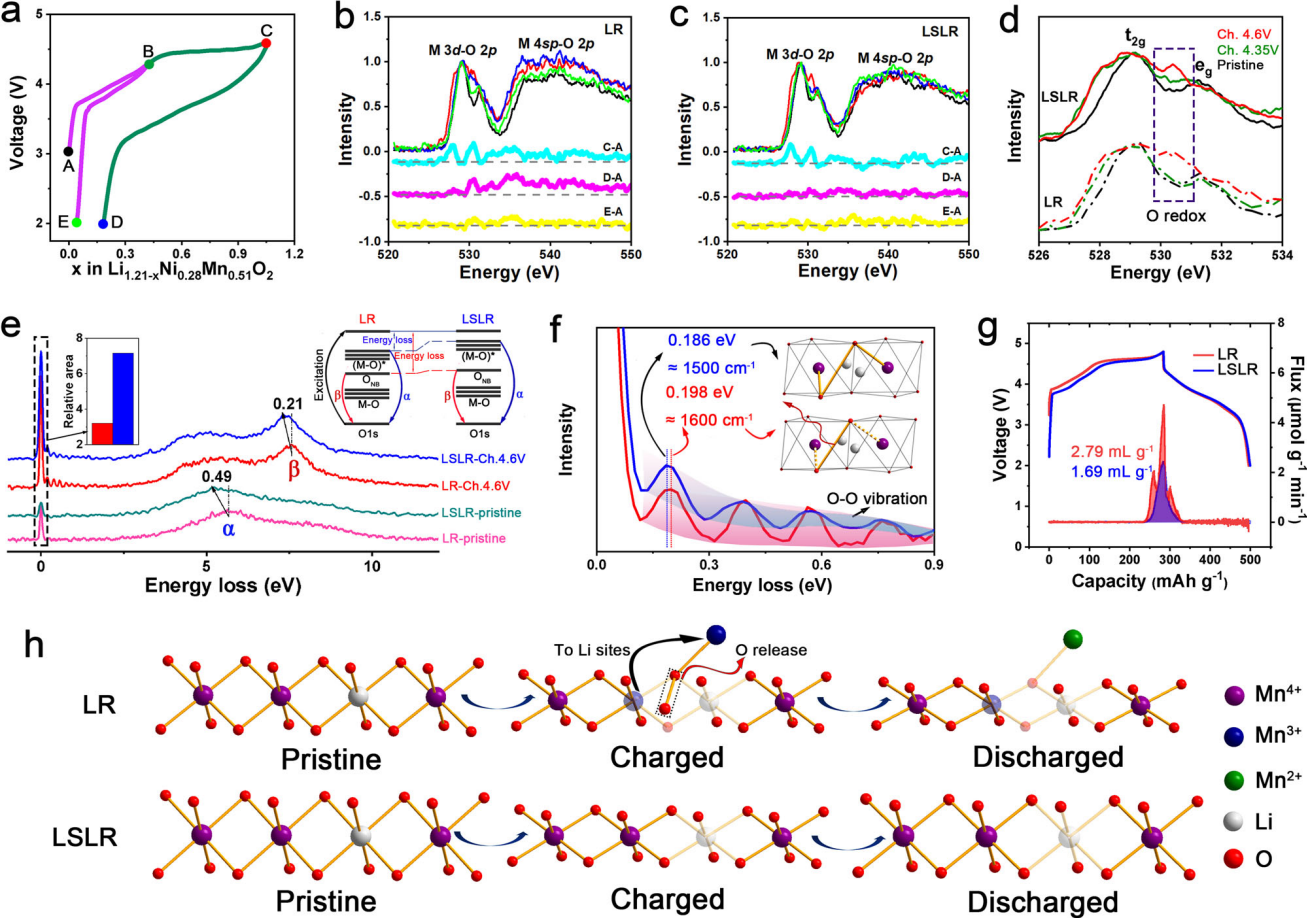
Oxygen redox behaviors in LR and LSLR. **a** The electrochemical state selection for collecting XAS spectrum. **b**, **c** The normalized O–K-edge spectra and the spectra difference between two designated points in the electrochemical curve for LR (**b**) and LSLR (**c**). Black represents the pristine state, dark green represents the 4.35-V charged state, red represents the 4.6-V charged state, blue represents the 2.0-V discharged state with a 4.6-V upper limit, and light green represents the 2.0-V discharged state with a 4.35-V upper limit. **d** The normalized pre-K-edge peaks of lattice oxygen. Ch. is short for charge. **e** The normalized O–K-edge RIXS for pristine and 4.6-V charged LR and LSLR electrodes. The insets declare the corresponding RIXS process and the relative elastic peak area of the two materials, setting the elastic peak area of each sample in the pristine state as 1. **f** Enlarged image in (**e**) at energy loss from 0 to 0.9 eV for the O–O vibration. The insets depict the schematic interaction between generated O–O species and Mn cations in bulk lattice. **g** Operando differential electrochemical mass spectrometry (DEMS) of O_2_ evolution rate during the initial cycle. **h** Sketch for illustrating cation migration and oxygen release in LR and LSLR. Source data are provided as a Source Data file.

To study the mechanisms of oxygen redox in LR and LSLR, synchrotron radiation soft X-ray techniques are adopted. The OAR properties of LR and LSLR at different states of charge are characterized by soft XAS, and the electrochemical state selection for the collecting spectrum is shown in Fig. 4a. Point A declares the open-circuit voltage (OCV) state. The voltages at Points B and C are 4.35 V and 4.6 V (vs Li^+^/Li), respectively, for separating the cationic and anionic redox reactions. D and E are the states of discharging to 2.0 V after charging to the upper limit of 4.6 and 4.35 V, respectively. Figure 4b, c exhibits the differentials between the (dis)charged and pristine state for the normalized O–K-edge spectra for LR and LSLR, so as to capture the distinction on OAR reactions between LR and LSLR. The C–A curves corresponding to LR and LSLR show similar fluctuations, indicating that OAR reaction occurs in both LR and LSLR at 4.6-V charged state. After the initial cycle with a voltage range of 4.6–2.0 V, the electronic structure of lattice oxygen for LR changed irreversibly, which is manifested by the spectra changes related with M 3*d*–O 2*p* and M 4*sp*–O 2*p*. While LSLR suppresses it well. When the voltage interval does not include OAR, the irreversible change of the electronic structure of lattice oxygen shown by cycled LR disappears, as displayed in curve E–A, indicating that the obvious fluctuations for the D–A line of LR are indeed caused by OAR, and it also proves the significant effect of LSLR in stabilizing OAR.

Figure 4d reflects the normalized pre-K-edge peaks of lattice oxygen in LR and LSLR. The spectra are normalized based on the t_2g_ peak at about 529 eV^31^. The peak at 530.5 eV is generally recognized as a characteristic peak of OAR^32^. At 4.35 V, the oxygen redox occurs in LSLR but not in LR, indicating a lower threshold potential and a different mechanism for OAR. The lower threshold potential of OAR is due to the more ionic O environments in LSLR^33^, which derives from the varied interactions of coordinated cations to lattice oxygen^34,35^. Studies show that the oxygen redox degree can be reflected by the integrating areas of pre-K-edge peaks of lattice oxygen^32,36^. It manifests that the lattice oxygen redox degree of LR and LSLR is very close (Supplementary Fig. 18). But the oxidation products of the lattice oxygen and the interactions between the oxidized oxygen and Mn in bulk lattice for the two samples are obviously different. Resonant inelastic X-ray scattering (RIXS) has a high-energy resolution and can keenly detect oxygen redox behaviors. All the RIXS spectra were tested at the excitation energy of 531 eV. For the convenience of quantitative comparison, the RIXS spectra are normalized based on the inelastic peak α at energy loss of about 5 eV (absolute energy: 526 eV). Peak α reflects the electronic structure of the metallic band of the oxides, while peak β is the RIXS characteristic of OAR in Li-rich oxides. Compared with LR, α and β in LSLR show different degrees of shifts toward low-energy loss (Fig. 4e). Both metallic and anionic bands have undergone energy elevating, but the metallic band elevates more. This phenomenon has an important relationship with the effect of LSLR on the inhibition of cation migration and OAR modulation, which will be explained in detail in the following section.

By comparing the relative integral area of the elastic peak, the difference of the lattice oxygen oxidation state in two samples can be captured (Fig. 4e). Specifically, the oxidation products of the lattice oxygen in LSLR project more oxygen anions with an electron hole than LR. In a latest study, this effect was also achieved in a cathode material for sodium-ion batteries^37^. The underlying fine structure could be revealed by the vibration near the elastic peak at 0.1–0.9 eV (Fig. 4f)^26,38^. It is reported that the vibration frequency is closely related to the species of O–O dimer^26^. For charged LR, a progression of small energy-loss peaks related to the O–O vibrations with a frequency of 1600 cm^−1^ rightly matches that of molecular O_2_. In this case, it can be interpreted as localized O_2_ molecule with Mn–O bond fracture in the lattice, which is consistent with Bruce’s work^26^. While for charged LSLR, the vibrational frequency is reduced to 1500 cm^−1^, indicating a lattice-bonded O–O dimer with a strengthened interaction between O–O dimer and Mn in the lattice for charged LSLR compared with charged LR^39^. Therefore, a deduction can be obtained that redox-active O–Mn bond in LSLR is more stable than LR. More evidence will be provided in the following. Moreover, the production of O–O species is also obviously inhibited for LSLR, which is demonstrated by the intensity of vibration in RIXS spectra (Fig. 4f) and the ex situ Raman spectra of the charged electrodes (Supplementary Fig. 19). Combined with the above spectral results, it can be proved that the OAR mechanisms of LSLR and LR are different. In LR, most of the lattice oxygen is oxidized to localized molecular O_2_ with weak interaction with the lattice, while in LSLR, most of the lattice oxygen is oxidized to oxygen anion with a localized electron hole, and a small part of lattice oxygen is oxidized to O–O dimer with strong interaction with the lattice.

For investigating the oxygen release quantitatively, operando differential electrochemical mass spectrometry (DEMS) is carried out. At the initial cycle, the generated oxygen amount of LSLR is about only half that of LR, and the charging capacity when oxygen begins to escape in LSLR is about 10 mAh g^−1^ more than that in LR. This confirms the results of RIXS and Raman, indicating that changing the oxidation products of lattice oxygen from localized O_2_ molecule to lattice-bonded O–O dimer or lattice oxygen with an electron hole is beneficial to restrain the lattice oxygen escape.

Based on the above discussions, a schematic is proposed (Fig. 4h). The behaviors of OAR will directly affect cation migration and oxygen release. For LR, lattice oxygen has been demonstrated to oxidize by forming localized O_2_ molecule. During the reaction, the distance between the active lattice oxygen anions decreases and O–O bridge bond is generated, with breaking the Mn–O bond in the MnO_6_ octahedron. For improving the thermodynamic stability of the damaged MnO_6_ octahedron, cation disorder in the form of Mn_Li_–V_Mn_ antisite-cation-vacancy point-defect pairs is created^40,41^. In the oxidized state, due to the instability of Mn–O chemical bond between lattice Mn and localized O_2_ molecule, electrons from Mn cations are grasped by localized O_2_ molecule, resulting in the reduction of Mn cations and lattice oxygen escape. This result has also been observed in other studies^31,42–44^. While for LSLR, localized O_2_ molecule is changed to lattice-bonded O–O dimer or lattice oxygen with an electron hole, and the destruction of Mn–O bonds is largely reduced, with maintaining MnO_6_ octahedron and the superlattice well during the (dis)charging process. Therefore, cation mixing and O_2_ generation, which are caused by Mn migration to the Li site, formation of unstable localized O_2_ molecule, and irreversible charge transfer from Mn to O, are obviously inhibited. Thus, LSLR shows a good structural resilience during cycling.

### First-principle calculations of LR and LSLR

**Fig. 5:**
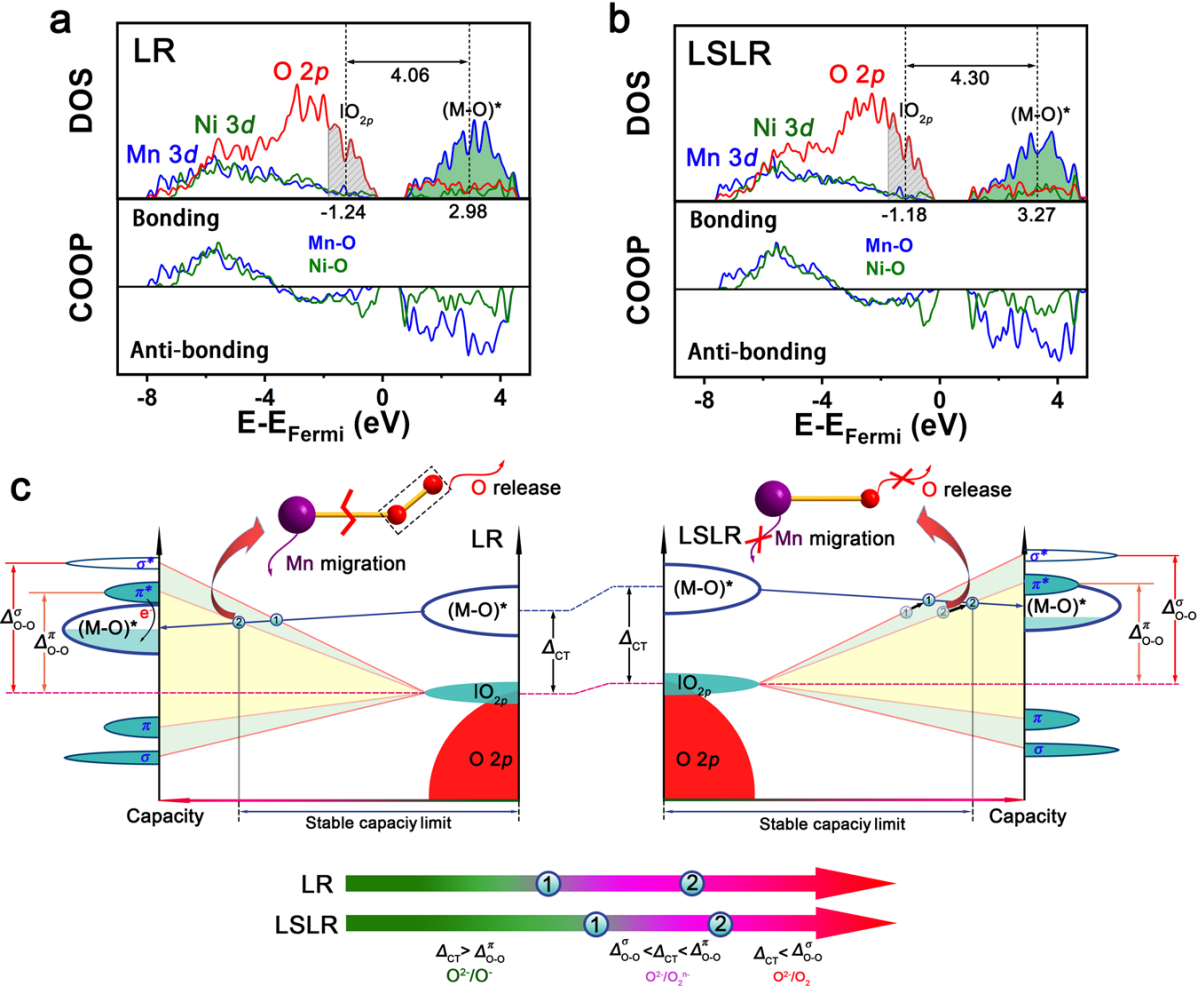
First-principle calculations of LR and LSLR. **a**, **b** Calculated partial DOS for O/Mn/Ni and COOP analysis of Mn–O/Ni–O in LR (**a**) and LSLR (**b**). **c** Sketch of the oxidation process in charge transfer on metallic and anionic bands for LR and LSLR. Source data are provided as a Source Data file.

First-principle calculations declare that the introduction of Zr into the lattice of LR has obvious effects on the characteristics of subtle crystal structure and the energy bands related with lattice O and Mn. First, compared with Li_2_MnO_3_ without Zr, the average Mn–O bond length in Li_2_MnO_3_ with Zr introduced was elongated (Supplementary Fig. 20). This has also been demonstrated by neutron diffraction refinement of LR and LSLR. The elongated Mn–O bond would increase the ionicity of the material, thus to increase the bandgap between the metallic and anionic states. In the dynamics aspect, this bandgap could also be called the charge-transfer gap (Δ_CT_). This signifies the energy that electrons have to overcome to transfer from the metallic band to the oxygen lone-pair band. Mathematically, Δ_CT_ shows the difference value between the band centers for antibonding M–O ((M–O)*) band and ׀O_2*p*_ band. Here, M stands for Ni and Mn. Declared by Fig. 5a, b, at energy range of 0–5 eV in the densities of states (DOS) of M *3d* orbitals, according to the crystal orbital overlap population (COOP), M–O is at antibonding state. For the energy range of 0 to −9 eV, the DOS of O 2*p* orbitals depict the M–O band and O 2*p* lone-pair state. Through calculating the band centers of (M–O)* and ׀O_2*p*_, Δ_CT_ could be obtained. Supplementary Fig. 21 declares, within 0 to −1.83 (or −1.74) eV in the DOS of O 2p for LR (or LSLR), roughly 1.36 electrons per LR (or LSLR) formula can be extracted. In all, 1.36 is exactly the number of electrons in ׀O_2*p*_ band for active O. Based on this, the band centers of ׀O_2*p*_ band for active O in LR and LSLR are calculated to be 1.24 and 1.18 eV, respectively. By stacking up the DOS of Ni *3d* orbitals and Mn *3d* orbitals, the energy band center of (M–O)* can be obtained after calculating (Supplementary Fig. 22). Compared with LR, both ׀O_2*p*_ and (M–O)* bands shift to higher energy locations in LSLR, and the (M–O)* bond elevates more, which accords with the RIXS results. It declares the expansion of Δ_CT_ after introducing Zr for LSLR, verifying the above deduction. Comparing with the calculated DOS diagrams for O 2*p* and Mn 3*d* orbitals of LR and LSLR before and after charge, the change of LSLR is less than that of LR (Supplementary Figs. 23 and  24). Furthermore, a signal caused by the O 2*p* splitting appears at the (M–O)* band range in the O 2*p* DOS diagram for charged LR, but it is not present for charged LRLS (Supplementary Fig. 23). This is consistent with the experimental results mentioned above.

## Discussion

On the basis of a previous study^45^, and our experimental and calculated results, we summarize a universal law to improve the reversibility and stability of OAR (Fig. 5c). With delithiating, the oxygen lone-pair band will split into two bonding states (*σ* and *π*) and two antibonding states (σ* and π*), and the metallic band will move down. When the metallic band overlaps σ* state, the oxidation product of lattice oxygen will transform from oxygen holes to peroxides. When the metallic band shifts further and reaches π* band, charge transfers will occur between π* and metallic bands, resulting in O_2_ formation and M cation reduction. While for LSLR, this situation is obviously suppressed by widening the bandgap and delaying the overlap of the metallic and anionic bands, which stabilizes redox-active O–M bond, anchors M cations and oxidized O anions simultaneously, thus fundamentally solving the issues of irreversible cation migration and O release.

Here, we appeal to researchers to concentrate on modulating OAR chemistry and paying more attention to the correlations between the metallic and anionic bands, with aiming at fundamentally addressing the critical issues of cathode materials with oxygen redox, such as lattice oxygen escape, voltage decay, irreversible cation reduction, and other daunting challenges.

In this work, we propose a strategy of modulating OAR chemistry to fundamentally address voltage fade by precisely constructing Li_2_ZrO_3_ slabs into Li_2_MnO_3_ domains in Li-rich Mn-based oxides through a simple and economical precipitation–calcination route. The introduction of Li_2_ZrO_3_ slab into Li_2_MnO_3_ domain, owing to the lattice strain, slips the Mn_2_Li slabs of Li_2_MnO_3_ ~3°, elongates Mn–O bond from 1.918 to 1.946 Å, resulting in the chemical environment changing of the active lattice oxygen and the bandgap widening between the metallic and anionic states. Experimental and calculated results reveal that the modulated band structure of Li-rich oxide expands the region in which lattice oxygen contributes capacity by oxidation to oxygen holes, delays the surpassing of the anionic state above the metallic state, and the charge transfer from anionic band to (M–O)* band, thus stabilizing the active lattice O–M chemical bond, restrains the formation of localized O_2_ molecule and the irreversible cation migration and reduction, fundamentally addressing the structural recession and voltage fade. This study provides a universal theory concerned with OAR and cation migration and explains the divergence on the correlation between OAR and cation migration. Furthermore, the stabilized high-capacity and low-voltage fade rate delivered by LSLR promises to build next-generation Li-ion batteries. The proposed insights on the importance of energy band modulation in restraining the formation of localized O_2_ molecule and migration of TM cations are applicable to other cathode materials with anionic redox.

## Methods

### Synthesis

To synthesize LR (Li_1.21_Ni_0.28_Mn_0.51_O_2_), stoichiometric amounts of NiSO_4_·6H_2_O (99.9%, MACKLIN), MnSO_4_·5H_2_O (99%, MACKLIN) were dissolved in distilled water and coprecipitated by H_2_C_2_O_4_·2H_2_O (99.5%, MACKLIN). The precipitated oxalate precursor was collected and washed with distilled water three times and with ethanol twice. After drying the precursor, it was mixed with 5% excess stoichiometric amount of LiOH·H_2_O (99%, MACKLIN) and calcined at 900 °C for 12 h in air.

To synthesize LSLR (Li_1.21_Ni_0.28_Mn_0.49_Zr_0.02_O_2_), stoichiometric amounts of NiSO_4_·6H_2_O (99.9%, MACKLIN), MnSO_4_·5H_2_O (99%, MACKLIN), and Zr(CH_3_COO)_4_ (Zr, 15.0–16.0%, MACKLIN) were dissolved in distilled water and coprecipitated by H_2_C_2_O_4_·2H_2_O (99.5%, MACKLIN). Refer to the synthesis of LR for the remaining steps.

### Characterization

The crystal structure of the as-prepared materials was characterized using an X-ray diffractometer (XRD, Ultima IV, Cu Kα) in 2*θ* range of 10 − 80°. Data were recorded at a step width of 0.02° and a scan rate of 10° per minute. Neutron powder diffraction measurements were performed on the powder diffractometer GPPD at China Spallation Neutron Source. The patterns were analyzed by the Rietveld refinement method using the software of FullProf. NMR data were collected on a 400-MHz Bruker Avance III spectrometer at the ^7^Li Larmor frequency of 155.13 MHz. The ^7^Li spectra were externally referenced with LiCl aqueous solution at 0.0 ppm. An aberration-corrected scanning transmission electron microscope JEM ARM200F (JEOL, Tokyo, Japan) equipped with two CEOS (CEOS, Heidelberg, Germany) probe aberration correctors was used to probe the structure at the atomic scale. The surface composition analysis was performed by X-ray photoelectron spectroscopy (XPS, Thermo escalab 250Xi) and Raman spectra (Renishaw inVia, 532 nm). Morphology of the samples was observed with a scanning electron microscope (SEM, HITACHI SU8010). Chemical compositions of the materials were measured by inductively coupled plasma optical emission spectrometer (ICP-OES, Agilent 730). Hard XAS was performed at BL14W1 beamline at Shanghai Synchrotron Radiation Facility (SSRF). Wavelet transform of XAS spectra is performed based on script (http://perso.u-pem.fr/farges/wav/).

### Electrochemical measurements

The cathode materials (80 wt%), such as carbon black (10 wt%) and polyvinylidene fluoride (10 wt%) were mixed uniformly with *N*-methyl pyrrolidinone. The obtained slurry was spread uniformly on an Al foil current collector and dried under vacuum at 110 °C for 12 h. The active material loading of the cathode electrode is 2‒3 mg cm^−2^. LiPF_6_ (1 M) dissolved in a mixture of ethylene carbonate (EC), dimethyl carbonate (DMC), and ethyl methyl carbonate (EMC) (1:2:2 by volume) was used as electrolyte. A polypropylene membrane with micropores (Celgard 2500) was used as the separator. The 2025-type coin cells were assembled in an Ar-filled glove box. Galvanostatic charge–discharge cycling was performed on a computer-controlled battery testing system (Neware, CT-4008T) at room temperature. The galvanostatic intermittent titration technique (GITT) measurement was programmed to supply a constant current of 0.1 C for 15 min and a subsequent relaxation for 60 min on an activated cell. Unless otherwise stated, all the cells were tested at an electrochemical window of 2.0‒4.8 V (vs. Li/Li^+^).

### Ex situ and operando electrode analysis

#### Ex situ hard XAS

Mn K-edges of electrodes at different states of charge were conducted at BL14W1 beamline at SSRF. A double-crystal monochromator was used. Metal foil was measured before the XAS measurements to make sure the data be calibrated in case of any drift of the monochromator position. The electrodes were measured in a transmission mode. The XAS data were processed using the Athena software. The electrodes were prepared by first galvanostatic charging/discharging the cells to the target voltage at 0.1 C and then disassembling the cells in an Ar-filled glove box. The electrodes were washed repeatedly with DMC. The preparation of the electrodes for XAS, RIXS, Raman, and AC-STEM is the same as here referred.

#### Ex situ soft XAS

O K-edges, Mn L_3_-edges, and Ni L_3_-edges of electrodes were conducted at beamline 20 A at NSRRC and beamline 02B02 at SSRF. The L-edge of Mn, Ni, and the K-edge of O XAS were collected using surface-sensitive total electron yield (TEY) and bulk-sensitive total fluorescence yield (TFY) modes simultaneously at room temperature in an ultrahigh vacuum chamber with a base pressure of ~5 × 10^−10^ Torr. The photon energy was calibrated with the spectra of the reference samples (MnO, NiO, and SrTiO_3_), which were measured simultaneously.

#### Ex situ RIXS

Ex situ RIXS data were collected at the PEAXIS beamline of synchrotron BESSY II at Helmholtz-Zentrum Berlin (HZB). The O K-edge RIXS spectra of the electrodes were collected at an excitation energy of 531.0 eV.

#### Ex situ Raman

Ex situ Raman experiment was performed on a Raman spectrometer. The prepared electrodes were sealed in a closed die with a quartz window.

#### Ex situ XRD

Ex situ XRD experiment was performed on a X-ray diffractometer. The electrodes were prepared through a linear voltammetry route on an electrochemical workstation 0.1 mV s^−1^.

#### Ex situ AC-STEM

The material was scraped off the prepared electrodes and ground gently in a mortar. Ethanol was added to disperse the sample, followed by ultrasonic treatment. The sample was then dropped onto the microgrid for TEM experiment. During sample preparation, the sample is strictly isolated from air.

#### In situ DEMS

In situ DEMS data were carried out using a cell with gas inlet and outlet ports. Ar carrier gas was flown at a constant rate (0.2 ml min^−1^) through the cell and into a quadrupole mass spectrometer (OmniStar GSD 320) equipped with a turbomole cular pump (Pfeiffer Vacuum).

### First-principles calculations

The density functional theory (DFT) calculations were performed using the Vienna Ab-initio Simulation Package (VASP)^46^. The generalized gradient approximation (GGA) with the Perdew–Burke–Ernzerhof (PBE)^47–49^ functional was used. Uniform G-centered k-point meshes with 2*π* × 0.04 Å^−1^ resolution and Methfessel–Paxton electronic smearing were used on the conformation in the Brillouin zone for geometric optimization. A cutoff energy of 500 eV was adopted for the simulation. Structure relaxation proceeded until all forces on atoms were less than 1 meV Å^−1^ and the total stress tensor was within 0.01 GPa of the target value, as in a previous study^50^. In order to accurately describe the electronic structure of the transition metal, the DFT + U approach was used, assuming *U* values for Mn-*3d*, Ni-*3d*, and Zr-*4d* of 6.0, 7.4, and 4.0 eV, respectively. For crystal orbital overlap population (COOP) calculations, the LOBSTER program^51^ was adopted.

## Supplementary information

Supplementary Information

## Data Availability

The data that support the findings of this study have been deposited at https://figshare.com/s/557bec3b0f938531343b. All other relevant data are available from the corresponding author on reasonable request. Source data are provided with this paper.

## Source data

Source Data

## Code availability

The authors declare that all the relevant codes are available within the paper and its Supplementary Information file or from the corresponding author upon reasonable request.
