# Supplementary material for: Addressing voltage decay in Li-rich cathodes by broadening the gap between metallic and anionic bands
Source: Nat Commun. 2021 May 24;12:3071. doi: 10.1038/s41467-021-23365-9 (PMC8144552; doi:10.1038/s41467-021-23365-9)
Supplement: Supplementary file 1 — Supplementary Information [file 41467_2021_23365_MOESM1_ESM.pdf]

Supplementary Information

Addressing voltage decay in Li-rich cathodes by broadening the gap between metallic and anionic bands

Jicheng Zhang[1], Qinghua Zhang[2], Deniz Wong[3], Nian Zhang[4], Guoxi Ren[4], Lin Gu[2], Christian Schulz[3], Lunhua He[2,5,6], Yang Yu[1] and Xiangfeng Liu[1,7*]

[1] Center of Materials Science and Optoelectronics Engineering, College of Materials Science and Optoelectronic Technology, University of Chinese Academy of Sciences, Beijing 100049, P. R. China

[2] Beijing National Laboratory for Condensed Matter Physics, Institute of Physics, Chinese Academy of Science, Beijing 100190, P. R. China

[3]Department of Dynamics and Transport in Quantum Materials, Helmholtz-Center Berlin for Materials and Energy, Hahn-Meitner-Platz 1, D-14109 Berlin, Germany

[4]Shanghai Institute of Microsystem and Information Technology, Chinese Academy of Sciences, Shanghai 200050, P. R. China

[5]Songshan Lake Materials Laboratory, Dongguan 523808, P. R. China

[6]Spallation Neutron Source Science Center, Dongguan, 523803, P. R. China

[7]CAS Center for Excellence in Topological Quantum Computation, University of Chinese Academy of Sciences, Beijing 100190, China

*liuxf@ucas.ac.cn

Supplementary Figures

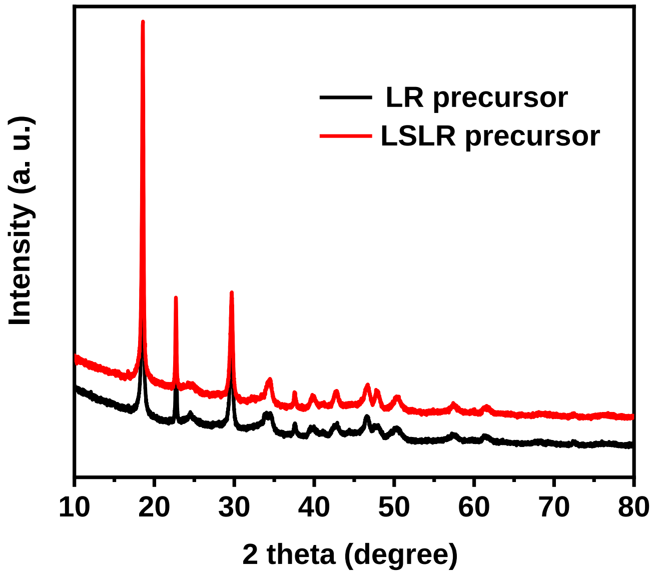

Supplementary Figure 1. XRD plots of synthesized precursors. Source data are provided as a Source Data file.

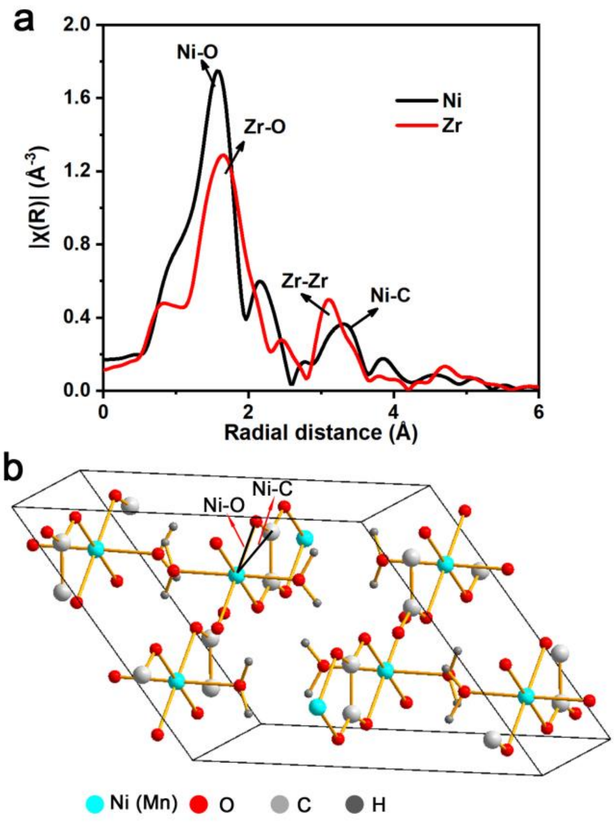

Supplementary Figure 2. Structure of LSLR precursor. a, Fourier transformed Ni/Zr K-edge EXAFS of LSLR precursor. **b**, Crystal structure for NiC_2O_4•$2H_2O$. The nearest two shells of the central Ni in LSLR precursor are Ni-O at ~2.0 Å and Ni-C at ~3.5 Å, which is consistent with the structure of NiC_2O_4•$2H_2O$. But Zr in LSLR precursor is demonstrated to have different shells, as illustrated by Supplementary Figure 3. Source data are provided as a Source Data file.

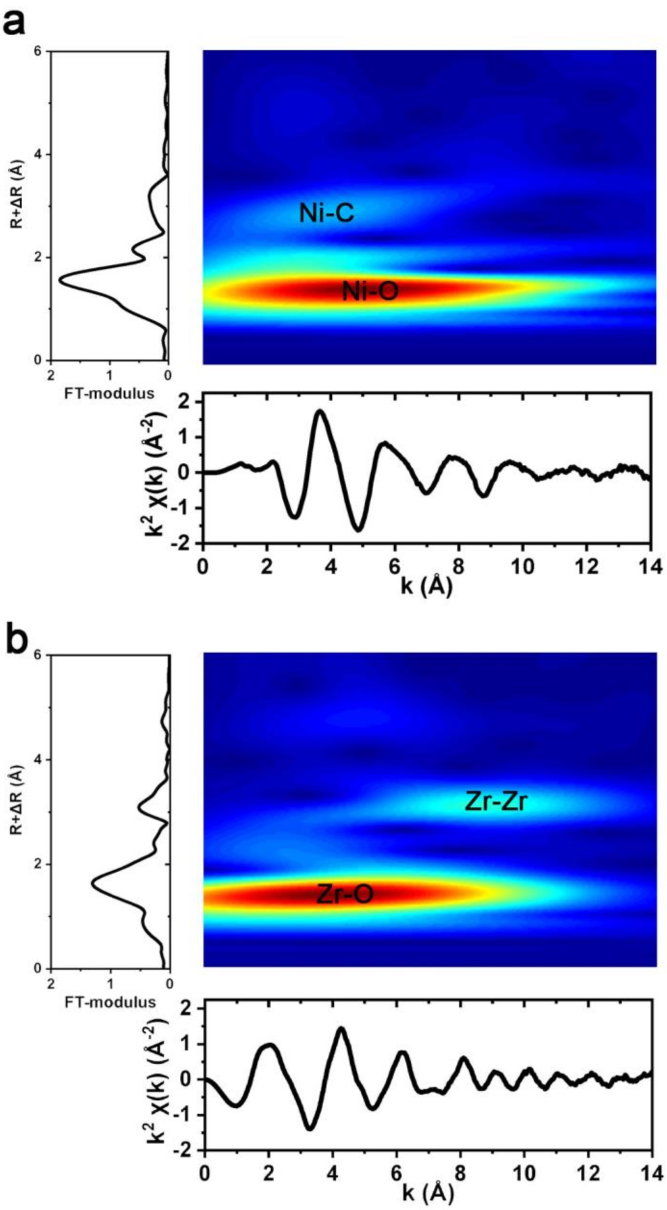

Supplementary Figure 3. Wavelet transform of XAS spectra of LSLR. a, Wavelet transform of Ni K-edge EXAFS. **b**, Wavelet transform of Zr K-edge EXAFS. This indicates that the first shell of Ni in LSLR precursor is Ni-O and the second shell is Ni-C, but the first shell of Zr in LSLR precursor is Zr-O and the second shell is Zr-Zr. This result verifies the structure of LSLR precursor shown in Fig. 1a in the text. Source data are provided as a Source Data file.

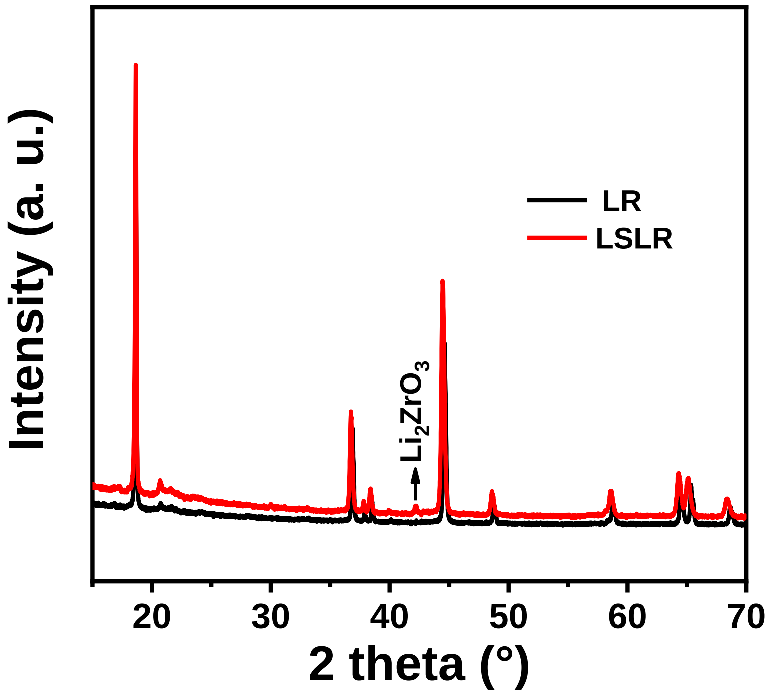

Supplementary Figure 4. XRD plots of prepared LR and LSLR powder. Source data are provided as a Source Data file.

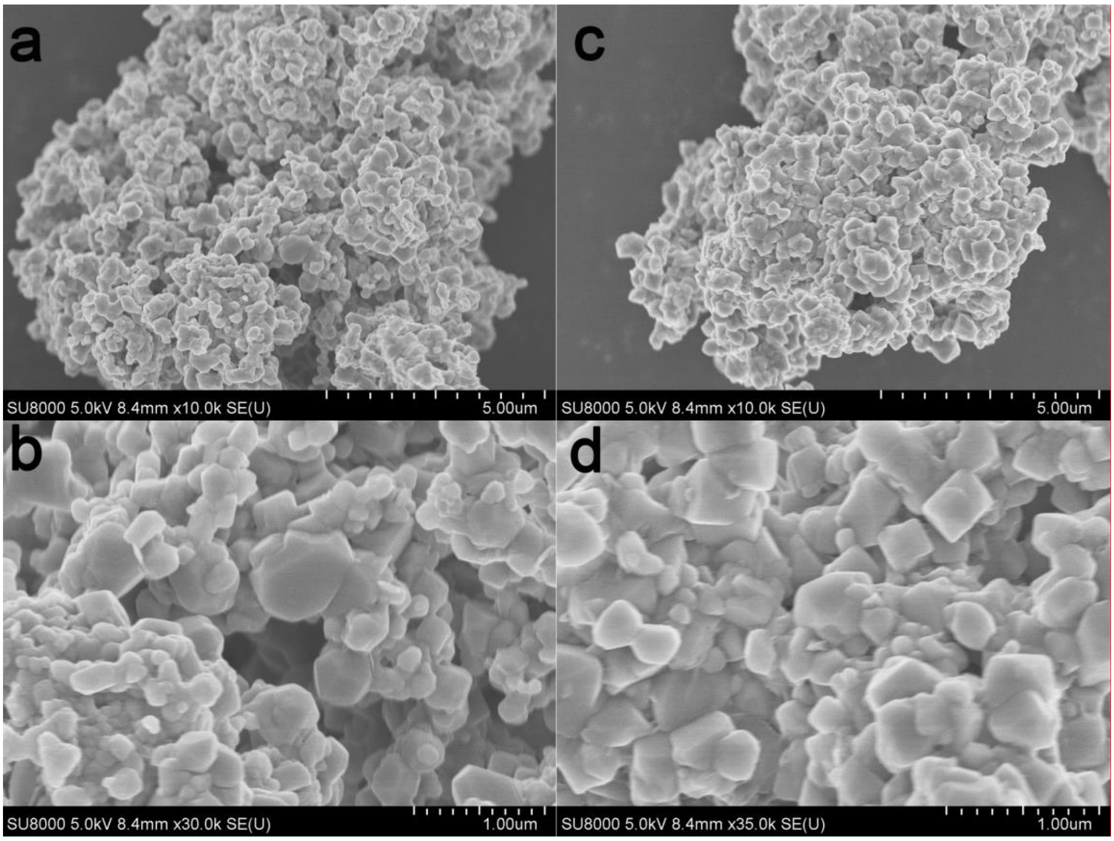

Supplementary Figure 5. Morphology of synthesized materials. a, b, SEM images of LR at low magnification (a) and high magnification (b). **c, d,** SEM images of LSLR at low magnification (c) and high magnification (d). Source data are provided as a Source Data file.

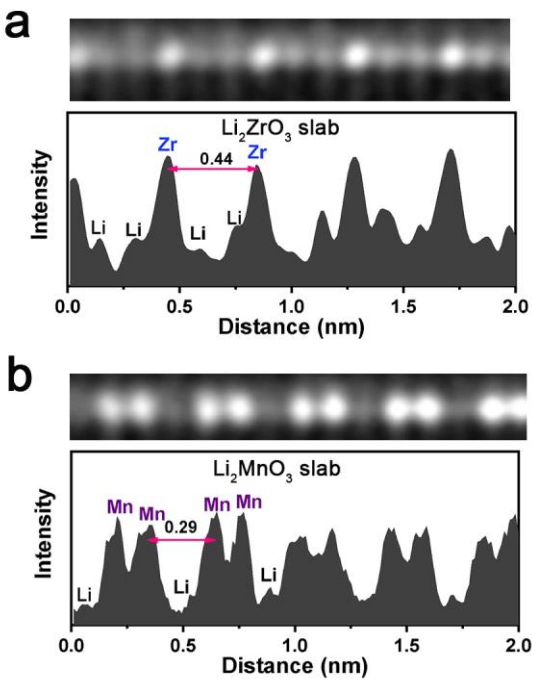

Supplementary Figure 6. Line intensity profiles of AC-STEM image. a, b, Line intensity profiles for Li_2ZrO_3 slab (a) and Li_2MnO_3 slab (b). Source data are provided as a Source Data file.

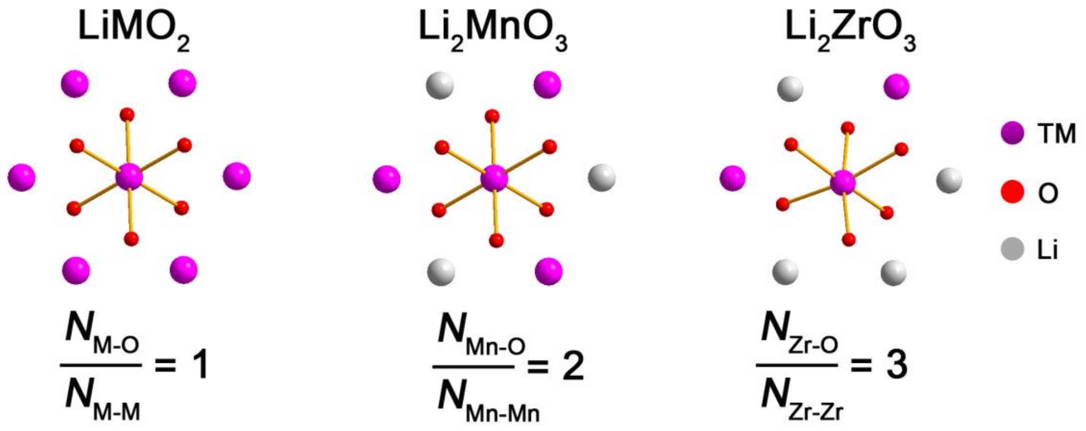

Supplementary Figure 7. Coordination for centered TM (M, Mn, Zr) in the ideal $LiMO_2$, Li_2MnO_3, Li_2ZrO_3 structure. Source data are provided as a Source Data file.

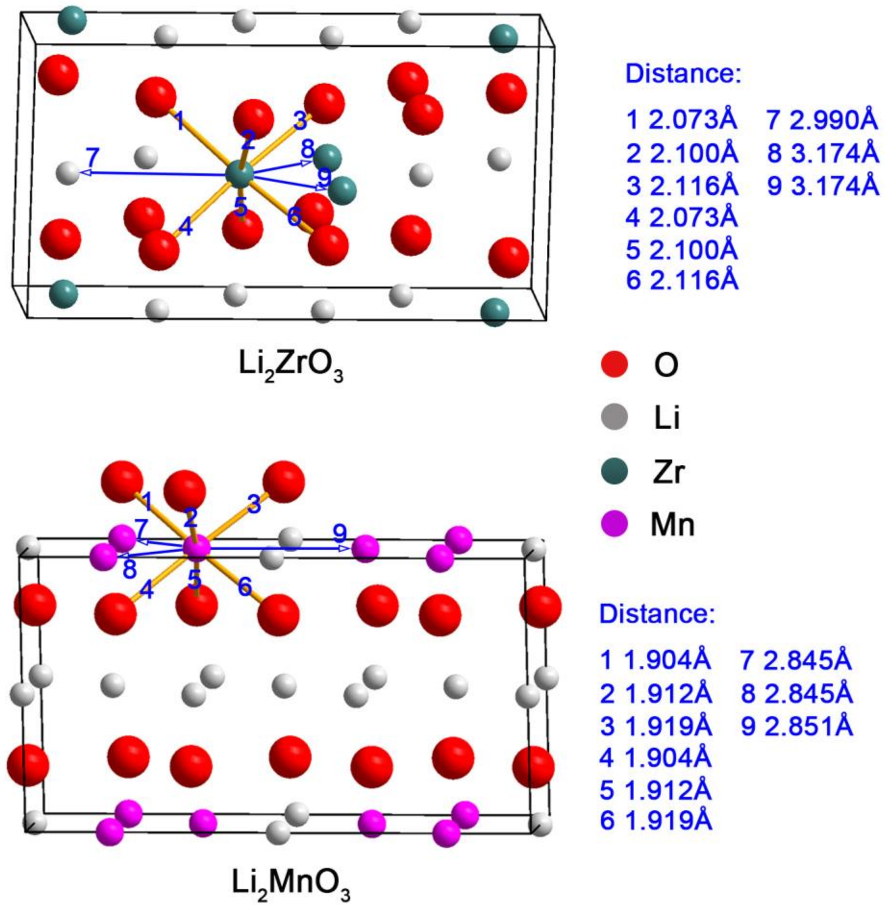

Supplementary Figure 8. The crystal information for Li_2ZrO_3 and Li_2MnO_3. Source data are provided as a Source Data file.

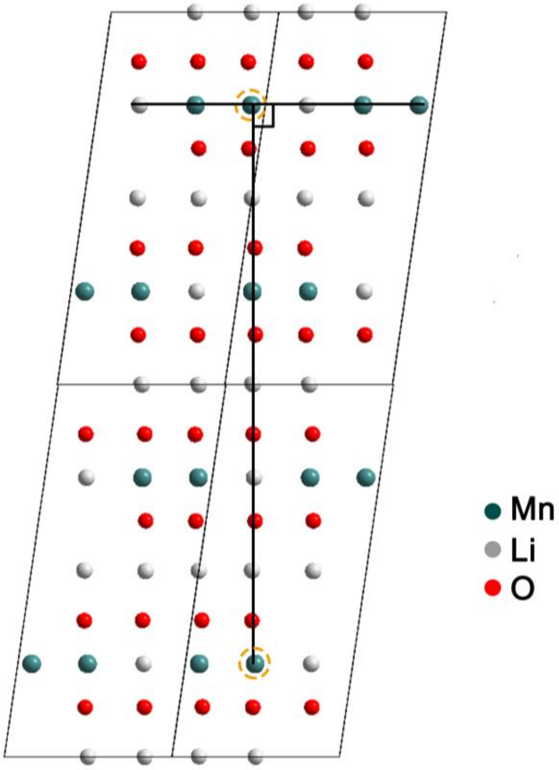

Supplementary Figure 9. Atom arrangement of the Li_2MnO_3 domain in LR. The Mn_2Li layers in this structure overlap perfectly every three Mn_2Li layers. Source data are provided as a Source Data file.

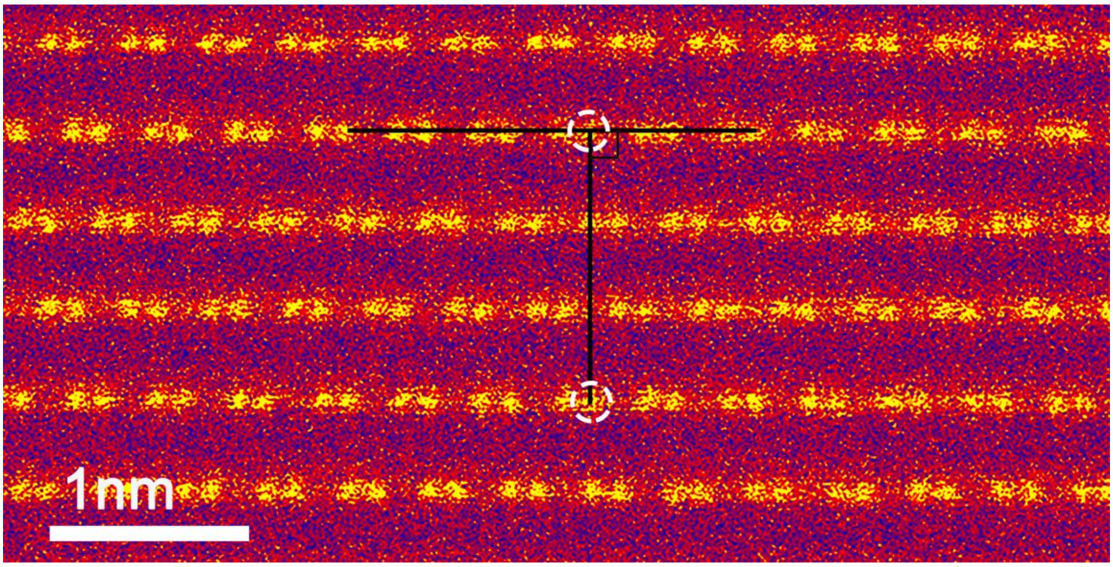

Supplementary Figure 10. AC-STEM image of Li_2MnO_3 domain in LR observed along the [110] axis. Source data are provided as a Source Data file.

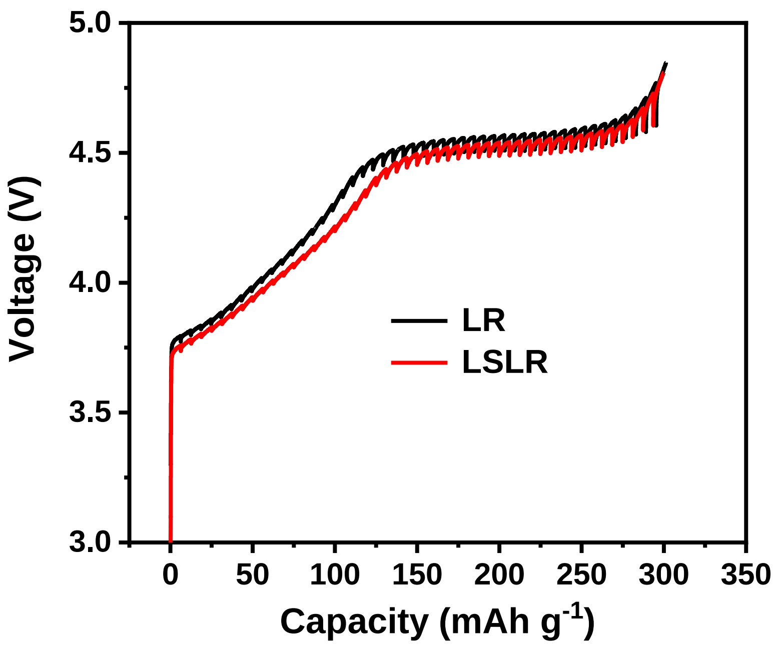

Supplementary Figure 11. The galvanostatic intermittent titration (GITT) plots. Li^+ diffusion coefficient is calculated through the equation: $D_{Li^+} = \frac{4}{\pi\tau}\left(\frac{m_B V_m}{M_B S}\right)^2\left(\frac{\Delta E_s}{\Delta E_\tau}\right)^2$, where τ, m_B, V_m, M_B and S respectively stand for the time duration during the current pulse, active material mass on the electrode, molar volume of active material, molecular weight of active material, and the contact area between the electrolyte and the electrode. ΔE_s is the difference in the steady-state voltage at a single-step GITT experiment, and ΔE_τ is the potential change during charging or discharging at the time of current of flux after subtracting the IR drop. Source data are provided as a Source Data file.

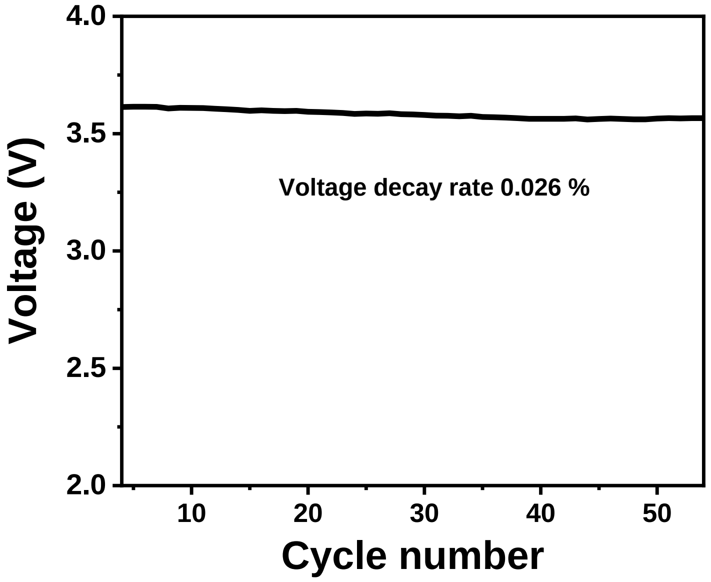

Supplementary Figure 12. The average voltage versus cycle number tested at 0.2 C for LSLR. Source data are provided as a Source Data file.

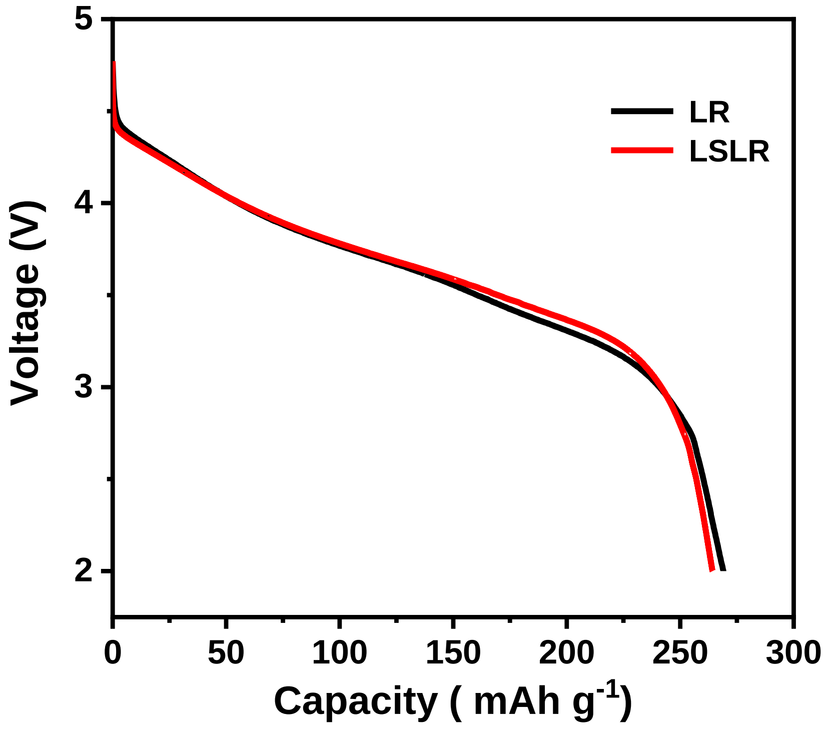

Supplementary Figure 13. The discharge plots for LR and LSLR at 0.05 C. Source data are provided as a Source Data file.

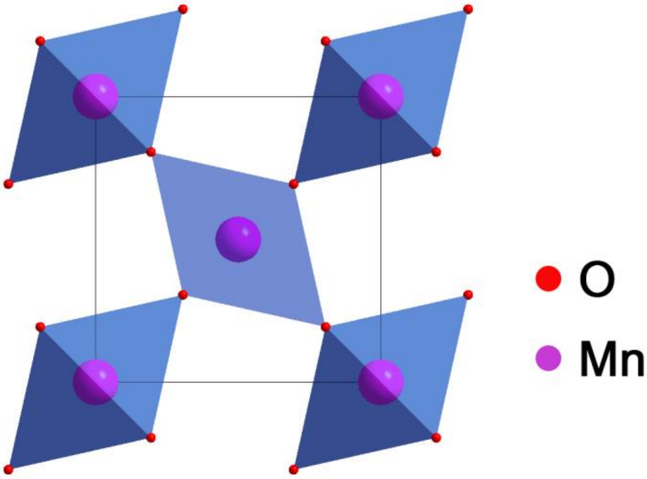

Supplementary Figure 14. The crystal structure for MnO_2 (Rutile, P42/mnm). Source data are provided as a Source Data file.

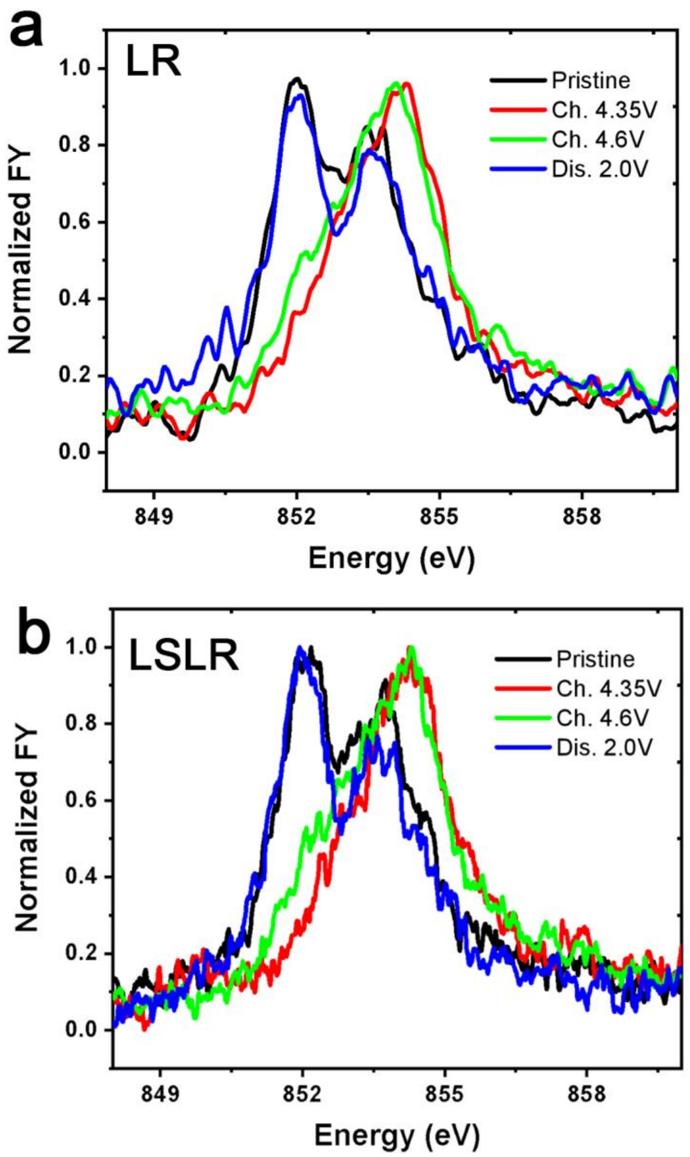

Supplementary Figure 15. Ni L_3-edge evolution at (dis)charging for LR (a) and LSLR (b). Source data are provided as a Source Data file.

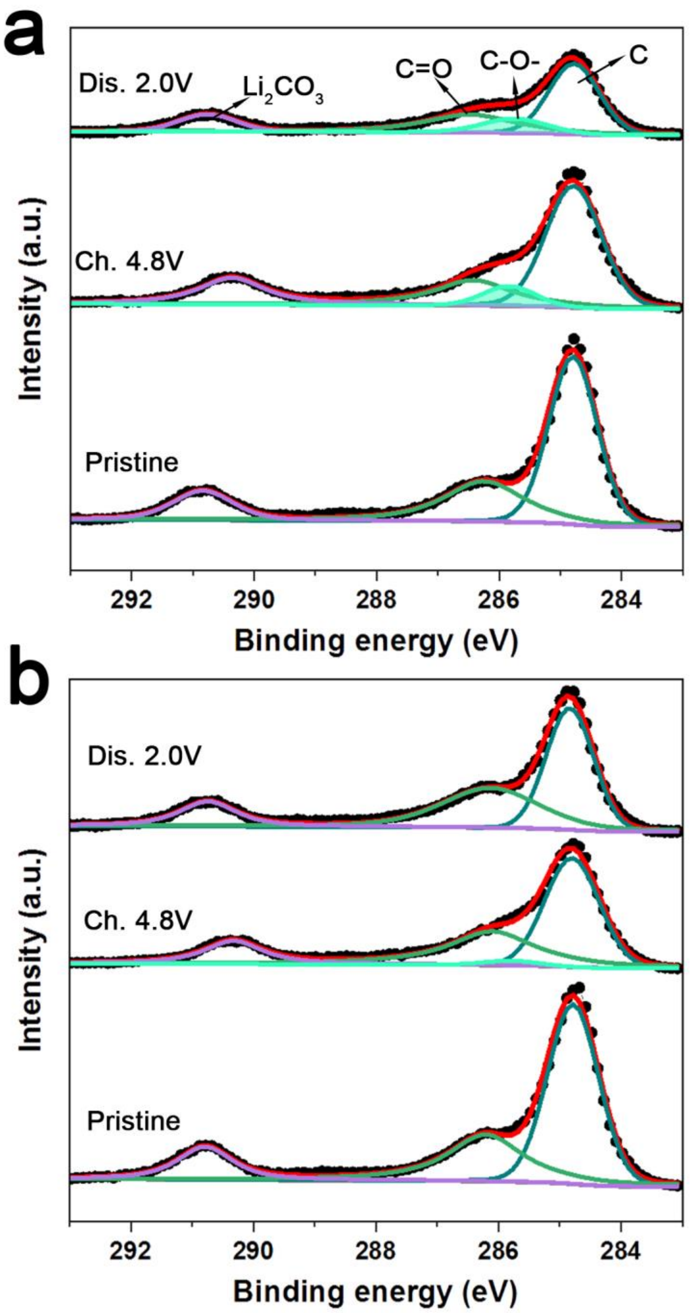

Supplementary Figure 16. *Ex-situ* C1s XPS spectra for LR (a) and LSLR (b). Source data are provided as a Source Data file.

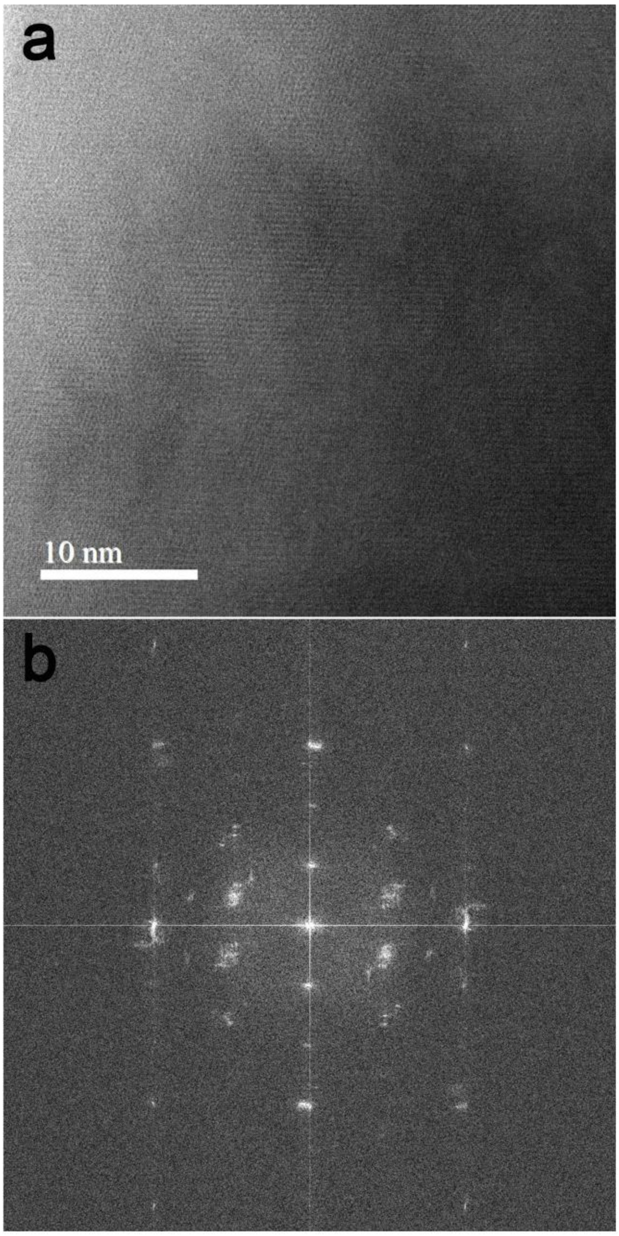

Supplementary Figure 17. AC-STEM image (a) and FFT image (b) for 4.6 V charged LR electrode. Source data are provided as a Source Data file.

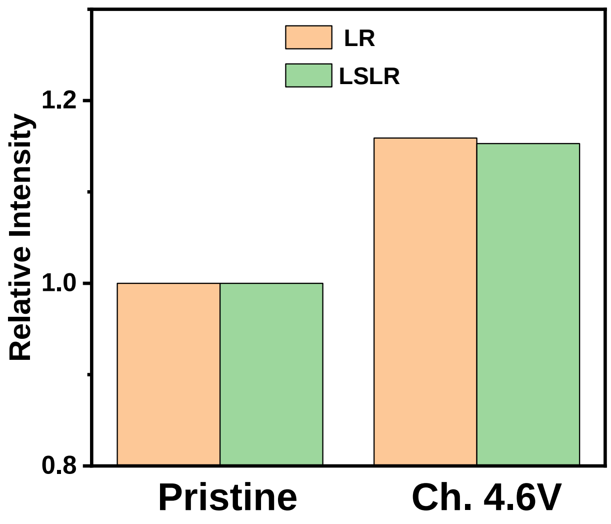

Supplementary Figure 18. The comparison for integrating area of O pre K-edge peaks in LR and LSLR. Source data are provided as a Source Data file.

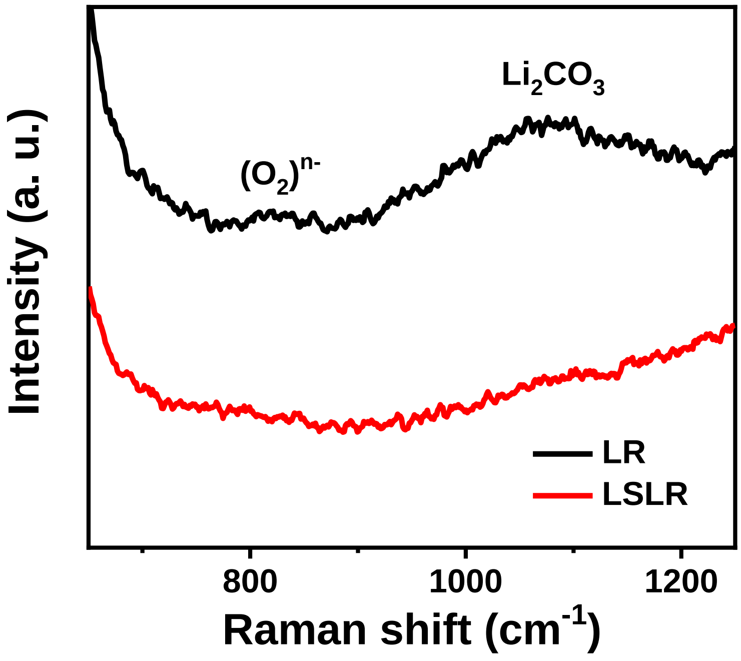

Supplementary Figure 19. Raman spectra of the 4.6 V charged electrodes of LR and LSLR. Source data are provided as a Source Data file.

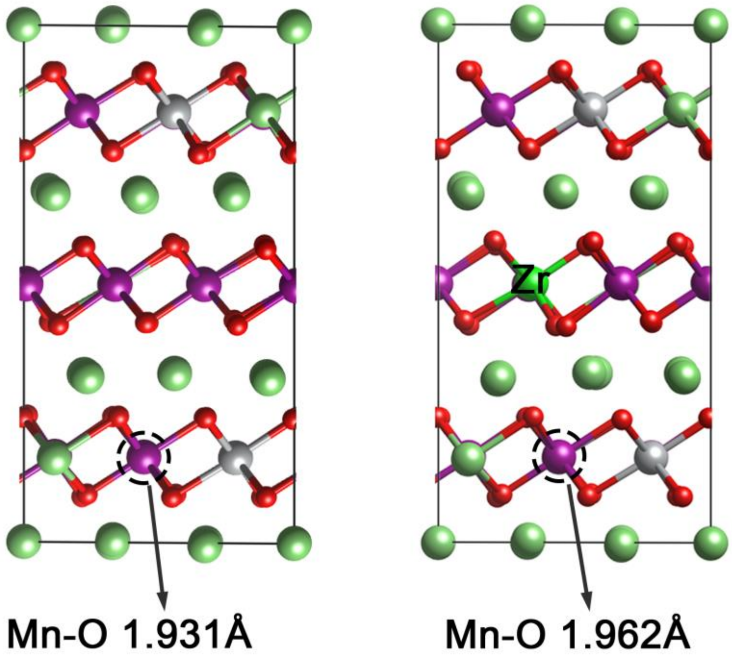

Supplementary Figure 20. Calculated crystal structure of Li_2MnO_3 before and after introducing Zr. Source data are provided as a Source Data file.

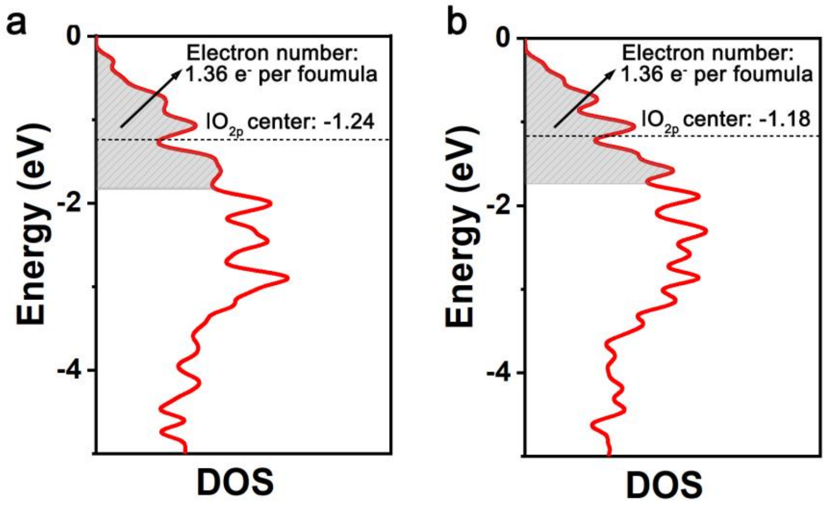

Supplementary Figure 21. DOS of the O 2p orbitals in LR (a) and LSLR (b). 1.36 is obtained by analyzing the model formula of LR ($Li(Li_{0.17}Ni_{0.27}Mn_{0.56})O_2$) or LSLR ($Li(Li_{0.17}Ni_{0.27}Mn_{0.5}Zr_{0.06})O_2$) used for DFT calculations. 1.17 Li can be removed from LR or LSLR, which need 1.17 electrons to compensate the charge balance. In addition to the 0.49 electrons extracted by Ni ($Ni^{2.2+} \rightarrow Ni^{4+}$), O can provide 0.68 electrons per formula. Since the oxygen anionic redox is the transition between O^{2-} and O^{1-}, the number of electrons in the $\text{l}O_{2p}$ orbital for the active O is 0.68×2=1.36.

$$\text{l}O_{2p}\text{ band center} = \frac{\int_{A}^{0} E \bullet \mathrm{DOS} \bullet \mathrm{d}E}{\int_{A}^{0} \mathrm{DOS} \bullet \mathrm{d}E}$$

. *E* is the abbreviation of energy. A is equal to -1.83 for LR and -1.74 for LSLR. Source data are provided as a Source Data file.

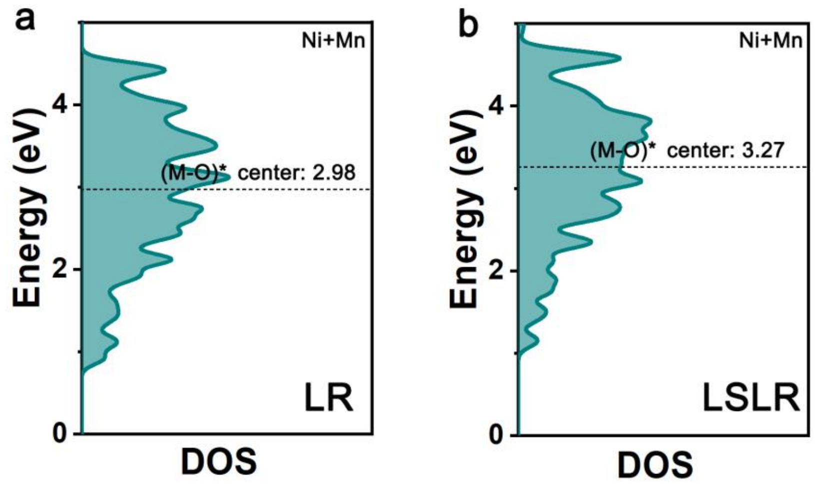

Supplementary Figure 22. DOS of the M 3d orbitals in LR (a) and LSLR (b). M stands for Mn+Ni. (M–O)* band center $= \frac{\int_0^5 E \cdot \mathrm{DOS} \cdot \mathrm{d}E}{\int_0^5 \mathrm{DOS} \cdot \mathrm{d}E}$. E is the abbreviation of energy. Source data are provided as a Source Data file.

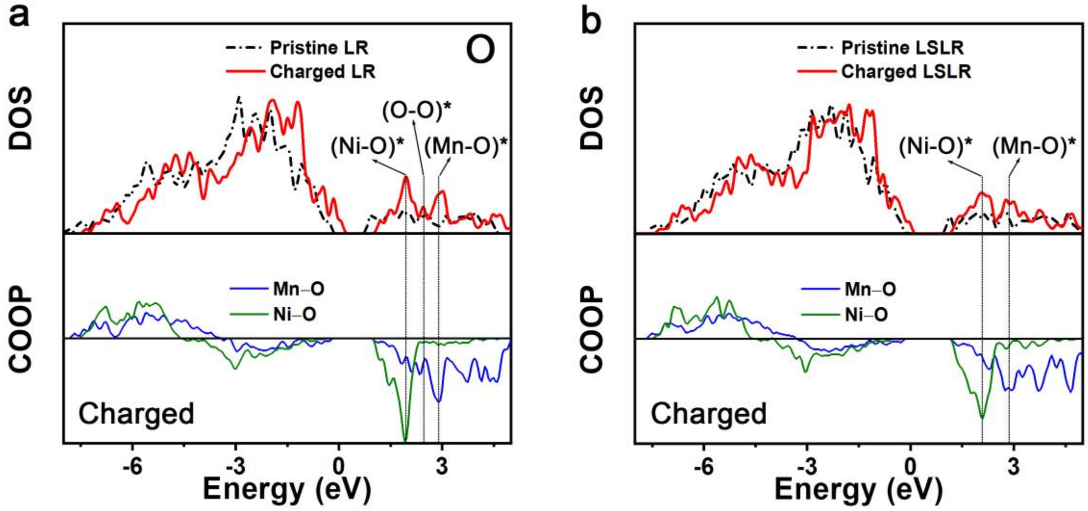

Supplementary Figure 23. DOS of the O 2*p* orbitals at pristine and charged states and COOP analysis of Mn–O/Ni–O at charged state for LR (a) and LSLR (b). Source data are provided as a Source Data file.

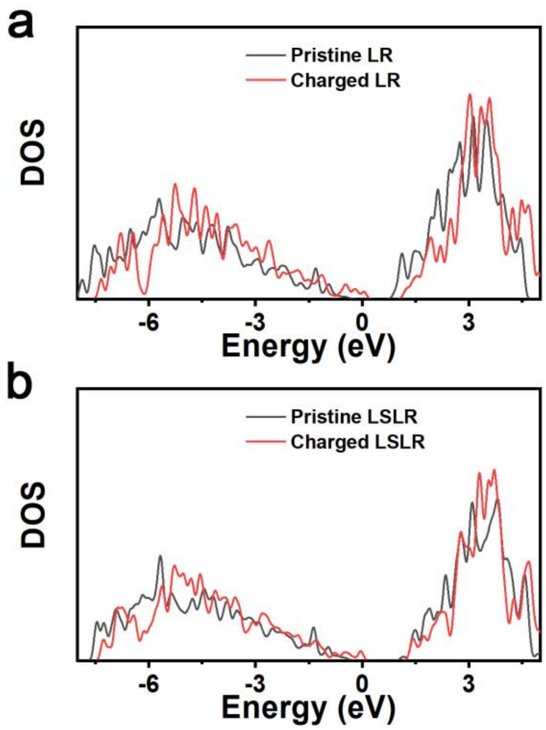

Supplementary Figure 24. Calculated Mn DOS of LR (a) and LSLR (b) before and after charge. Source data are provided as a Source Data file.

Supplementary Tables

Supplementary Table 1. Elemental ratio of LR and LSLR derived from ICP-OES. Source data are provided as a Source Data file.

Samples	Li	Ni	Mn	Zr
LR	1.21	0.28	0.51	_
LSLR	1.21	0.28	0.49	0.02

Supplementary Table 2. Cell parameters, Li/Ni disorder derived from neutron diffraction Rietveld refinement. Source data are provided as a Source Data file.

Samples	a (Å)	c (Å)	z_{ox}	$S_{(MO2)}$ (Å)	$I_{(LiO2)}$ (Å)	Li/Ni disorder (%)
LR	2.7902(3)	13.8956(18)	0.2584(8)	2.0825	2.5494	3.4
LSLR	2.8297(4)	14.0885(20)	0.2583(6)	2.1142	2.5819	3.2

$S_{(MO2)} = 2(1/3-Z_{ox})\times c$ $\quad$ $I_{(LiO2)} = c/3-S_{(MO2)}$

Supplementary Table 3. The voltage-decay rate comparison between our sample and others with modification.

Materials	Voltage decay per cycle (mV)	Measurement condition	Reference
Ti^{4+} doped Li-rich oxide	4.21	120 cycles, 2.0 − 4.8 V, 24.2 mA g^{-1}, RT	1
F^{-} doped Li-rich oxide	3.20	100 cycles, 2.0 − 4.8 V, 50 mA g^{-1}, RT	2
O2 type Li-rich oxide-1	1.43	50 cycles, 2.0 − 4.8 V, 80 mA g^{-1}, RT	3
O2 type Li-rich oxide-2	~1.1	40 cycles, 2.0 − 4.8 V, 5 mA g^{-1}, RT	4
Lithium deficiencies Li-rich oxide	~5.80	500 cycles, 2.0 − 4.8 V, 250 mA g^{-1}, RT	5
Li-gradient Li-rich oxide	1.17	200 cycles, 2.0 − 4.8 V, 50 mA g^{-1}, RT	6
Li/Ni disordered Li-rich oxide	1.33	70 cycles, 2.0 − 4.8 V, 50 mA g^{-1}, RT	7
Three-in-one Li-rich oxide	1.09	500 cycles, 2.0 − 4.8 V, 200 mA g^{-1}, RT	8
Polyacrylic acid treated Li-rich oxide	1.04	100 cycles, 2.0 − 4.8 V, 100 mA g^{-1}, RT	9
LSLR	0.95[a]	50 cycles, 2.0 − 4.8 V, 50 mA g^{-1}, RT[a]	This work
	0.45[b]	300 cycles, 2.0 − 4.8 V, 250 mA g^{-1}, RT[b]	

Supplementary References

1. Yu, Z., Shang, S.-L., Gordin, M. L., Mousharraf, A., Liu, Z.-K. & Wang, D. Ti-substituted $Li[Li_{0.26}Mn_{0.6-x}Ti_xNi_{0.07}Co_{0.07}]O_2$ layered cathode material with improved structural stability and suppressed voltage fading. *J. Mater. Chem. A* **3**, 17376–17384 (2015).
2. Li, L. et al. Retarded phase transition by fluorine doping in Li-rich layered $Li_{1.2}Mn_{0.54}Ni_{0.13}Co_{0.13}O_2$ cathode material. *J. Power Sources* **283**, 162–170 (2015).
3. Zuo, Y. et al. A high-capacity O2-type Li-rich cathode material with a single-layer Li_2MnO_3 superstructure. *Adv. Mater.* **30**, 1707255 (2018).
4. Eum, D., et al. Voltage decay and redox asymmetry mitigation by reversible cation migration in lithium-rich layered oxide electrodes. *Nat. Mater.* **19**, 419–427 (2020).
5. Liu, P., et al. Lithium deficiencies engineering in Li-rich layered oxide $Li_{1.098}Mn_{0.533}Ni_{0.113}Co_{0.138}O_2$ for high-stability cathode. *J. Am. Chem. Soc.* **141**, 10876–10882 (2019).
6. Zhu, Z. et al. Gradient Li-rich oxide cathode particles immunized against oxygen release by a molten salt treatment. *Nat. Energy* **4**, 1049–1058 (2019).
7. Zhang, J. et al. Tuning oxygen redox chemistry in Li-rich Mn-based layered oxide cathodes by modulating cation arrangement. *Adv. Mater.* **31**, 1901808 (2019).
8. Ding, X., Luo, D., Cui, J., Xie, H., Ren, Q. & Lin, Z. An ultra-long life Li-rich $Li_{1.2}Mn_{0.6}Ni_{0.2}O_2$ cathode by three-in-one surface modification for lithium-ion batteries. *Angew. Chem. Int. Ed.* **59**, 7778–7082 (2020).
9. Yang, J. et al. Suppressing voltage fading of Li-rich oxide cathode via building a well-protected and partially-protonated surface by polyacrylic acid binder for cycle-stable Li-ion batteries. *Adv. Mater.* **10**, 1904264 (2020).
